# Work-family trajectories across Europe: differences between social groups and welfare regimes

**DOI:** 10.3389/fsoc.2023.1100700

**Published:** 2023-11-30

**Authors:** Mustafa Firat, Mark Visser, Gerbert Kraaykamp

**Affiliations:** Department of Sociology, Radboud University, Nijmegen, Netherlands

**Keywords:** work, family, life course, gender, welfare regime, sequence analysis, SHARE

## Abstract

**Introduction:**

Work and family trajectories develop and interact over the life course in complex ways. Previous studies drew a fragmented picture of these trajectories and had limited scope. We provide the most comprehensive study of early-to-midlife work-family trajectories to date.

**Methods:**

Using retrospective data from waves 3 and 7 of the Survey of Health, Aging and Retirement in Europe (SHARE), we reconstructed work-family trajectories from age 15 to 49 among almost 80,000 individuals born between 1908 and 1967 across 28 countries. We applied multichannel sequence and cluster analysis to identify typical trajectories and multinomial logistic regression models to uncover their social composition.

**Results:**

The results revealed six common trajectories. The dominant and therefore standard trajectory represents continuous full-time employment with having a partner and children. Women, the lower educated and persons from conservative and liberal welfare regimes are underrepresented in this trajectory, whereas men, higher educated people and those from social-democratic, Eastern European and Baltic welfare regimes are overrepresented. The other trajectories denote a deviation from the standard one, integrating a non-standard form of work with standard family formation or vice versa. Mothers in a stable relationship generally work part-time or not at all. When mostly in full-time employment, women are more likely to be divorced. Lower educated persons are less likely to have work-family trajectories characterized by full-time work and a non-standard family, yet more likely to be non-employed for large parts of their life with standard family formation. Younger cohorts are underrepresented in non-employment trajectories, but overrepresented in part-time employment trajectories along with a partner and children as well as full-time employment trajectories with divorce. Individuals from Southern European and liberal regimes are more likely to be non-working and self-employed partnered parents and those from social-democratic regimes are more likely to be full-time employed divorced parents. We also found pronounced gender differences in how educational level, birth cohort and welfare regime are associated with work-family trajectories from early to midlife.

**Discussion:**

Our findings highlight the socially stratified nature of earlier-life work-family trajectories in Europe. Potential implications for inequalities in later life are discussed.

## 1 Introduction

Work and family are central to our lives. Many of us feel content to have a job, a partner or children. Yet, our roles as workers, partners and parents do not always reconcile. Life course events and transitions in one domain can motivate or force us to in- or decrease our involvement in another domain. For example, a person may postpone family formation to establish a career (Aassve et al., 2007), a full-time employed person may switch to part-time employment upon becoming a parent (Biemann et al., 2012) or a divorced person may return to employment after being non-employed to compensate for the lack of a partner's financial support (Struffolino et al., 2020). As exemplified in these situations, how we arrange our work life is not independent from how we arrange our family life (and vice versa). Therefore, it is essential to understand how work and family jointly evolve over an extended period of a person's life.

Understanding the joint development of work and family lives is important because it informs us about social inequality as the interdependency between work and family varies along social and structural lines (Fasang and Aisenbrey, 2021). Although some people enjoy the privilege of connecting work and family harmoniously, others face systematic barriers, with events in their work life restricting their family life (or vice versa). For instance, men and highly educated people tend to have more stable careers and partnerships because their work and family lives often sustain each other (McMunn et al., 2015; Madero-Cabib and Fasang, 2016). In contrast, the employment careers of women are usually interrupted by marriage and childbirth while less educated people's partnerships are more susceptible to dissolution due to job insecurity (Lu et al., 2017; Hogendoorn et al., 2022). Thus, life-long patterns of concurrent events and transitions in combined work and family lives, known as work-family trajectories, are socially stratified.

However, our knowledge of work-family trajectories and their social stratification is still limited (for a review, see Machu et al., 2022; Han and Mortimer, 2023). When examining work-family trajectories and their social composition, most studies singled out a specific gender (e.g., women) in a certain country (UK: Aassve et al., 2007; Spain: Davia and Legazpe, 2014). Studies including both men and women generally concentrated on a single country (Switzerland: Madero-Cabib et al., 2016; Germany: Engels et al., 2019; US: Fasang and Aisenbrey, 2021) or compared just two countries (McDonough et al., 2015; Aisenbrey and Fasang, 2017). When they included more countries and covered different welfare regimes, they again focused on a specific gender (e.g., women; Ice et al., 2020) or relied on relatively small sample sizes for some countries while also not examining how work-family trajectories differed across social groups other than men and women (Uccheddu et al., 2022). Only a few studies involved multiple social groups (e.g., based on gender, education and/or birth cohort) and multiple countries. Yet, these studies examined whether work and family trajectories have become more complex over time and to what extent complexity (i.e., a summary measure quantifying the number of states and transitions between states within a sequence) varies cross-nationally (Van Winkle and Fasang, 2021), but not how work-family trajectories unfold over the life course among different social groups and countries. When they did so, their focal point was restricted to earlier trajectories (e.g., until age 35) covering the transition to adulthood, but lacking information on work-family trajectories at older ages (Lesnard et al., 2016; Schwanitz, 2017). These limitations hamper the generalizability of existing research findings because they rest on data from particular social groups, life phases, historical times and institutional contexts.

In this study, we address these limitations and offer several contributions. First, we focus on a longer lifespan, more states defining work and family sequences and more individuals and countries than any prior study, thus better capturing the continuity, multiplicity and heterogeneity of work-family trajectories. We examine work-family trajectories from age 15 to 49, considering that these are the “prime ages” at which people shape their work-family life. We do so for almost 80,000 individuals across 28 European countries, covering nearly 2.8 million person-years between 1923 and 2017. Hence, we increase generalizability to a wider population. Second, we assess how these work-family trajectories from early to midlife are differentiated by gender, education, birth cohort and welfare regime. Unlike most studies, we look at these factors simultaneously, controlling the associations for each other (cf. Schwanitz, 2017). This informs us about the socially stratified nature of early-to-midlife work-family trajectories and how social inequalities unfold over the earlier life course. Third, we unravel how said work-family trajectories differ between men and women by education, birth cohort and welfare regime. Although the influence of these factors is considered to be gendered (Becker, 1981; Sainsbury, 1999; Bukodi et al., 2012), past work has paid inadequate attention to this, with narrower scope and thus generalizability (e.g., gender differences in family trajectories by education in Finland; Jalovaara and Fasang, 2015). Finally, we make our code producing the trajectory data publicly available, which may facilitate future research. The work-family trajectories from our study could be used in cross-national multilevel analyses to show the later-life outcomes of the earlier-life work-family trajectories, like retirement and wellbeing.

We use data from the 3rd (2008–2009) and 7th (2017) waves of the Survey of Health, Aging and Retirement in Europe (SHARE, Börsch-Supan et al., 2013). The SHARE data provide fine-grained retrospective and cross-national information suitable for answering our research questions: (1) How do people's early-to-midlife work-family trajectories look like across Europe and (2) to what extent are gender, educational level, birth cohort and welfare regime associated with these trajectories? To answer the first question, we employ the cutting-edge technique of multichannel sequence and cluster analysis, which is an ideal method for our purpose, as it accounts for interdependencies between multiple life domains and delivers holistic trajectories (Aisenbrey and Fasang, 2010). To answer the second question, we present average marginal effects to make the interpretation of the results based on multinomial logistic regression models more intuitive. As part of answering the second question, we also explore whether differences between educational levels, birth cohorts and welfare regimes in the work-family trajectories vary between men and women.

## 2 Theoretical notions

**Figure 1:**
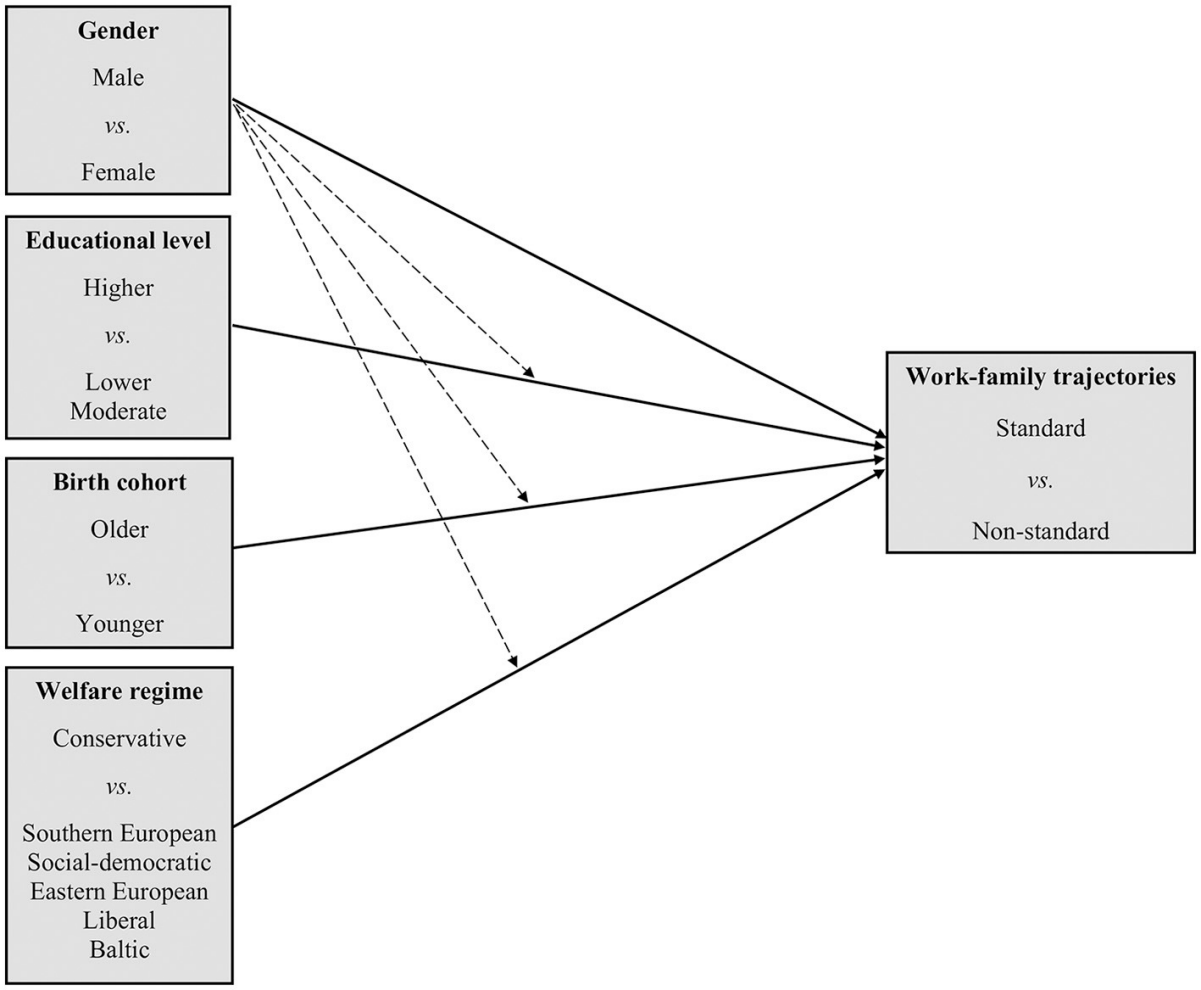
Conceptual model.

According to the life course perspective (Elder et al., 2003), the timing, order and duration of life events shape individual lives and work-family trajectories, which are contingent on socio-demographic characteristics (e.g., gender and education), historical time and the institutional and normative context. We follow this perspective and adopt a sequential approach to the study of work-family trajectories from early to midlife, allowing us “*to study a complex set of life course trajectories as they actually take place, providing ideal types of trajectories that can be interpreted and analyzed in a meaningful way*” (Aassve et al., 2007, p. 371). To make it easier to interpret and analyze trajectories in a meaningful way, we make a distinction between “standard” and “non-standard” trajectories. Here, “standard” refers to the most common trajectory, which has often been found to be continuous full-time employment combined with having a partner and children. “Non-standard” refers to a less prevalent trajectory, which is basically all trajectories other than the most common one, such as those involving self-employment, part-time employment, divorce or childlessness. While recognizing the drawbacks of this simplification, we believe that it provides a helpful conceptual structure^1^. Given the explorative nature of our approach, we do not formulate hypotheses on the number or content of the work-family trajectories. Yet, although we do not know a-priori which earlier-life work-family trajectories we will find, we assume that both standard and non-standard ones exist. Based on this general assumption, we argue who is more likely to be overrepresented in which type of trajectory. We also explore whether education, birth cohort and welfare regime relate differently to work-family trajectories for men and women. This implies that we empirically take a gendered perspective on life courses. Theoretically, however, we do not go into much detail about it and leave it more explorative. This is because there is a lack of theory and research on the intersection of gender with education, birth cohort and welfare regime when it comes to work-family trajectories. We hope that our research contributes to theory development on these intersections. Figure 1 illustrates our conceptual model.

### 2.1 Gender

Work-family trajectories are known to be gendered (Madero-Cabib and Fasang, 2016). A primary reason for this is the traditional male breadwinner and female homemaker norm. As a result of the church's role and a conservative political discourse, men were assumed to be (full-time) employed and provide for their families. In contrast, women were usually assigned the caregiver or housekeeper role, with non-employment and economic dependence on men throughout the marriage. While this norm dominated life courses in Western countries in the previous century and although it still is relevant, it has lost support owing to historical changes (e.g., secularization) and institutional reforms (e.g., childcare provision) (Trappe et al., 2015). Consequently, the convention of non-employment among (married) women has weakened and (part-time) employment has increased, also because of increasing educational attainment among women (Cunningham, 2008).

Women's family life has significantly changed with their increasing labor market participation. While pursuing a career, they delay family formation and remain single for longer than men (Aassve et al., 2007). Once in a partnership, they make more transitions between different partnership states than men, exhibiting more divorce (Van Winkle and Fasang, 2021). When they give birth, they often take a break from work or reduce working hours, but men's employment career is usually uninterrupted (Biemann et al., 2012; McMunn et al., 2015). If women want to rejoin the workforce after childbirth, they may have difficulties securing a job or they earn less than their pre-birth wage, facing the “motherhood penalty” (Lu et al., 2017).

Despite women's emancipation and policy efforts to support the dual-earner/dual-carer model, the male breadwinner and female homemaker norm persists. On the one hand, women are encouraged to join the workforce with initiatives like formal childcare services. On the other hand, they bear the labor market penalty of becoming a parent. This disparity can harm work-family reconciliation and foster non-standard work-family trajectories in women's early to midlife (McMunn et al., 2015). Thus, it is likely that women are overrepresented in non-standard while men are overrepresented in standard work-family trajectories over the earlier part of their life course. However, in the case of a non-standard trajectory involving self-employment, men are likely overrepresented in it, as women prefer non-employment and part-time employment over self-employment due partly to gender-based barriers to entrepreneurship (Verheul et al., 2012).

### 2.2 Educational level

Education is another factor shaping work-family trajectories. According to the theories of human capital (Becker, 1964), signaling (Spence, 1973), and segmentation (Piore, 1975), lower educated people have fewer opportunities in the labor market and generally acquire more insecure positions than higher educated people. Therefore, they encounter more unemployment (Visser et al., 2016a). They also receive less on-the-job training and develop fewer professional skills over their career, disposing them to be perceived as less productive than their higher educated counterparts (Cairó and Cajner, 2018). As these practices impair human capital accumulation in lower educated individuals, employers prefer hiring the higher educated. The lower educated are usually located in the secondary segment of the labor market, which offers jobs with lower status and pay (Gesthuizen et al., 2011), or they become part-time employed, mostly involuntarily (Cam, 2012). Moreover, they often work in physically demanding jobs under harsher conditions, increasing the risk of disability (Falkstedt et al., 2014). The labor market insecurity faced by lower educated individuals affects their family life. For example, it has repercussions for partnering dynamics (Scherer, 2009). Lower educated persons (and their partners) experience more life (e.g., economic) strains, which cause unstable partnerships (Hogendoorn et al., 2022), causing divorce and leading to single motherhood (Zimmermann and Konietzka, 2018). It also has repercussions for parenting practices. For instance, lower-educated couples engage less in childcare activities because of their employment conditions, especially when they have three or more children (Biegel and Maes, 2022). This implies that how couples combine and divide work and family responsibilities may be different for those who have higher or lower education (Visser and Fasang, 2018) in combination with having a few or several children.

Hence, it is likely that the lower educated are overrepresented in non-standard work-family trajectories, while the higher educated are overrepresented in standard work-family trajectories from early to midlife. However, considering that education offers unequal returns for men and women in the work and family domain (Becker, 1981), the role of education in earlier work-family trajectories might differ by gender. This is because the opportunity costs of remaining outside the workforce to get married and rear children are higher for higher educated women. They invest time and effort into higher education and thus have stronger incentives to work and delay family formation (Aassve et al., 2007). Contrastingly, it is in general less costly for lower educated women to engage in early family formation and leave the labor market (Berrington and Pattaro, 2014). So far, there has been scarce research testing such gender differences, which is important to address because it can illuminate the gendered nature of social stratification in early-to-midlife work-family trajectories.

### 2.3 Birth cohort

The destandardization hypothesis posits that life courses in Europe have become less institutionalized and orderly over time and, instead, more individualized and unpredictable (Brückner and Mayer, 2005). People would increasingly deviate from conventional life courses in the face of increasing options or constraints in life. Work-family trajectories transform across birth cohorts because each cohort grows up and enters adulthood under specific structural, institutional and cultural circumstances. It is assumed that older cohorts [i.e., those born before or during the Second World War (WWII)] grew up under harder life conditions, but started their career and formed their family under less unequal working conditions and more traditional family norms (Lesthaeghe, 2010; Crystal, 2018). These older cohorts established their career and family in the post-war era of economic expansion, industrialization and institutional and cultural support for marriage and fertility. However, younger cohorts (i.e., those born after WWII) entered the workforce and established a family when labor markets, social norms and gender roles were transforming (Lesthaeghe, 2010; Crystal, 2018). Hence, there might be differences in workforce participation and family formation between older and younger cohorts. For example, it is known that part-time jobs, temporary positions, self-employment, unmarried cohabitation and childlessness have become more common in the last decades due to globalization, labor market flexibilization and normative changes regarding marriage and fertility (Kalleberg, 2000; Zimmermann and Konietzka, 2018; Damman and Von Bonsdorff, 2021). Yet, there might also be differences within these broader groups. For instance, among older cohorts, those who experienced WWII as adults (e.g., born between 1900 and 1920) may differ from those who experienced it as teenagers or children (e.g., born between 1921 and 1940). The ones who experienced it as adults faced the immediate challenges of rebuilding and stabilizing their work and family lives, while the ones who experienced it as teenagers or children may have internalized different values and attitudes regarding work and family due to disruptions in their family structure and social environment.

This highlights the role of socialization as a mechanism that distinguishes cohorts with respect to their work-family trajectories. Older cohorts were socialized when part-time work was uncommon, female employment was perhaps unorthodox and marriage and parenthood were cherished. Younger cohorts were socialized when these work and family norms were shifting, which coincided with the improved position of women in society due to increased female schooling (Epstein et al., 2014). Changes in educational opportunities, gender roles and labor markets accelerated changes in people's values and attitudes regarding partnering and parenthood—a process known as the “second demographic transition” (Lesthaeghe, 2010). At the same time, family formation became less governed by religious institutions because of secularization (Studer et al., 2018). These transformations eroded the prevalence of traditional work and family arrangements, resulting in less social control or stigmatization of non-traditional work and family arrangements, such as part-time employment, self-employment and unemployment in the work domain and union dissolution, unmarried cohabitation and singlehood in the family domain (Biemann et al., 2012; Zimmermann and Konietzka, 2018). Yet again, there might be divergences within the broader groups of older and younger cohorts. For example, younger cohorts (e.g., born between 1945 and 1966) are often ascribed a tendency toward postmodern values, more divorce and a higher emphasis on education and career. However, there could be differences within this cohort depending on, for example, how influential their exposure to the 1969 movements was and whether they entered the labor market and parenthood in the late 1960s or the early 1980s.

Nevertheless, the influence of economic, normative and socialization processes on work-family constellations from early to midlife is expected to be most pronounced among those born in the 1970s and onwards, as they are the ones who underwent these processes the most (Lesnard et al., 2016). Given that the cohorts we observe in the SHARELIFE data were all born before that period, we acknowledge that it might be hard to establish cohort differences in earlier work-family trajectories (see Van Winkle and Fasang, 2021). Still, there is a tangible basis for cross-cohort heterogeneity, with the broader group of younger cohorts (i.e., born after 1945) being overrepresented in non-standard and the broader group of older cohorts (i.e., born until 1945) being overrepresented in standard work-family trajectories over the earlier part of their life course.

Cohort change in earlier work-family trajectories is plausibly different for men and women. The reason is that institutional reforms (e.g., parental leave and childcare provision), shifts in gender role attitudes and the service sector expansion are likely to have made more prominent changes in women's lives. In the past, women usually left the workforce after marriage or childbirth. Today, many women delay family formation to build a career, although part-time work and career breaks are still more common among women (McMunn et al., 2015). Such changes are likely less pronounced for men, resulting in more similar male work-family trajectories over the earlier life course (Van Winkle and Fasang, 2021). Despite this, prior work on work-family trajectories has largely ignored gendered cohort effects (e.g., Uccheddu et al., 2022).

### 2.4 Welfare regime

Alongside who one is and when one is born, life courses are also shaped by where one lives because the institutional context in countries, mainly their social policies, shape the individual life course by creating opportunities and constraints (Mayer, 2009). The notion of welfare regime proposes two organizing principles to capture and distinguish between countries' social policy contexts (Esping-Andersen, 1990, 1999) that are also central to work-family trajectories. One is decommodification, which refers to the degree to which the state protects people against labor market risks. The other is defamilization: the reduction of dependence on the family so that people can uphold a standard of living through paid work or the social security system. Although these two principles are useful to distinguish countries in terms of their welfare regime, they (especially decommodification) have been criticized by feminist scholars for being relatively blind to gender. Authors such as Orloff (1993), Leitner (2003), Bambra (2004), and Verbakel et al. (2023) have taken a gendered approach and suggested that countries should be classified into welfare regimes based on the extent to which they are familialistic and defamilialistic. In a familialistic state, the family is the main provider of care, which means that women are encouraged or expected to care for children and older people. On the contrary, in a defamilialistic country, the state is responsible for providing care; hence, it enables women to participate in the labor market by offering formal care services.

Empirical research categorizing countries by their level of decommodification, familialism and defamilialism has found similar clusters of welfare regimes, suggesting that differences in clusters result from the name given to a type of welfare regime rather than the actual grouping of countries (Gauthier and Koops, 2018). In fact, Bambra (2004) created a typology of welfare regimes based on the principle of familialism and compared it to Esping-Andersen (1990) typology based on the principle of decommodification, arriving at the following conclusion: “*The resulting typology has shown stark similarities with the ‘three worlds of welfare' typology and it therefore undermines the gender critique as it suggests that, whilst Esping-Andersen had gender in the corner rather than the fore front of his eye when constructing the decommodification index, it nonetheless has fairly accurately captured the extent to which women's experiences of the welfare state differ by country and regime type”* (Bambra, 2004, p. 209). This means that although the naming may differ across approaches, countries usually fall into the same groups in terms of their welfare regime type.

The literature has identified several welfare regime types, which differ in their level of decommodification, familialism and defamilialism. In conservative regimes (e.g., Germany), people in paid work are eligible for unemployment, sickness and pension benefits, indicating higher decommodification. However, these benefits usually strongly depend on prior earnings, with continuous full-time employment being rewarded. When it comes to the family domain, conservative regimes seem to be between familialistic and defamilialistic, since caring responsibilities are often attributed to women, although childcare provisions are also available. Yet, gender inequality in employment tends to be high in conservative regimes (Möhring, 2016).

Social-democratic regimes (e.g., Sweden) are more redistributive and progressive than conservative regimes, being more decommodified and more defamilialistic. They have universal social security systems and provide generous sickness, unemployment and pension benefits, regardless of prior earnings (Esping-Andersen, 1990, 1999). Next to employment protection, social-democratic regimes make childbearing less costly for employment careers through childcare provision and parental leave (Pezer, 2022). Moreover, they support the dual-earner model, where couples equally participate in the workforce. Therefore, women's labor market attachment is relatively strong in social-democratic regimes.

Liberal regimes (e.g., Ireland) are market-oriented, thus low on decommodification. There is a strong reliance on the market, which produces welfare. This welfare, however, is produced in a socially unequal way, partly because individuals bear the responsibility of labor market risks. Individuals with high incomes and prestigious occupational positions have easier access to welfare benefits and can, for instance, retire earlier or have a partner who may exit the workforce more easily (Komp-Leukkunen, 2019). Consequently, people in these regimes are more active in the workforce. Yet, this workforce participation is gender unequal, because these regimes encourage a traditional breadwinner-caretaker norm, similar to conservative regimes (Korpi et al., 2013).

Eastern European countries present an interesting case. Despite being different from conservative and social-democratic regimes, Eastern European countries can be expected to show similar work-family trajectories over the earlier life course (Aidukaite, 2009). This is particularly expected among our respondents, who were born before the 1970s and did not wholly experience the life course outcomes of the transition from communism to democracy (Van Winkle and Fasang, 2021). Under communist rule, the state owned the labor market and made employment mandatory, meaning that the state provided continuous full-time employment careers for all citizens. During that era, Eastern European countries also adopted pro-natalist policies that encouraged marriage and childrearing in combination with high decommodification by strict employment protection and gender-equal practices regarding caring (Van Winkle and Fasang, 2021).

However, it can be problematic to put all Eastern European countries into one type of welfare regime. There is probably heterogeneity among Eastern European countries in relation to employment careers and family arrangements (Möhring, 2016). This is because the process of their transition from communism to democracy has been different, potentially leading to different constellations. Specifically, as indicated by Van Winkle and Fasang (2021), countries from Central Europe and the Balkans are more similar to one another in that they are characterized by lower complexity in work-family trajectories, whereas countries from the Baltic region are more different, characterized by higher complexity. This suggests distinguishing between Eastern European regimes (covering Central and Balkan countries, such as Poland and Bulgaria) and Baltic regimes (e.g., Latvia) will be more informative than lumping all Eastern European countries together (cf. Uccheddu et al., 2022).

Southern European regimes (e.g., Spain) provide fewer means for decommodification and defamilialism, thereby representing familialistic welfare states. Welfare provisions in these regimes are fragmented and not interventionist. Individuals have limited access to welfare benefits because of lower public expenditures on social security programs (Ferrera, 1996). Thus, people rely largely on family support to deal with negative labor market forces and use their own resources to raise a child, sometimes at the expense of their career stability (Gough, 1996). This basically implies that women usually take the responsibility of childcare and elderly care on their shoulders (Möhring, 2016), paving the way for a high gender inequality in labor market participation (Schmitz et al., 2023).

Overall, it can be argued that higher decommodification, lower familialism and higher defamilialism foster standard, while lower decommodification, higher familialism and lower defamilialism foster non-standard work-family trajectories in earlier life. Hence, it is plausible that people from liberal and Southern European regimes are overrepresented in non-standard earlier-life work-family trajectories. People from conservative, social-democratic, Eastern European and Baltic regimes are likely overrepresented in standard earlier-life work-family trajectories, although Baltic regimes may appear slightly more in non-standard trajectories relative to other Eastern European countries. Yet, the costs of certain non-standard life courses may be lower when decommodification and defamilialism are higher. For example, part-time work is more established in conservative and social-democratic regimes, where part-timers are paid at the same rate per hour as full-timers and are entitled to pro-rata social benefits (Bosch, 2004). Part-time jobs are thus more attractive in conservative and social-democratic regimes, allowing for work-family reconciliation.

Again, the role of welfare regimes in shaping work-family trajectories from early to midlife is gender-specific as policies are gendered. Policies can be more relevant for women because welfare regimes differ in their regulatory potential for women with incentives to work while forming a family (Sainsbury, 1999). For example, social-democratic regimes promote dual-earners/dual-carers through public childcare provisions and parental leave policies for both men and women. Women in these countries are therefore expected to be more likely to follow a standard work-family trajectory from early to midlife than women in conservative regimes, where policies in place support the male breadwinner model. Yet, only a few studies have examined such dynamics by comparing work-family trajectories across different regimes for a large number of countries (Komp-Leukkunen, 2019; Schmitz et al., 2023), drawing an incomplete picture. Furthermore, research has mostly contrasted the US with European countries (McDonough et al., 2015; Van Hedel et al., 2016), restricting our knowledge on European countries. This knowledge gap is valuable to address for understanding how cultural norms (e.g., attitudes on female employment) and institutional contexts (e.g., parental leave and childcare provision) that are embedded in welfare regimes function differently for men's and women's work-family lives.

## 3 Methods

### 3.1 Data

We used data from the 3rd and 7th wave of SHARE, called SHARELIFE (Börsch-Supan, 2022a,b). To handle these data, we adapted the Stata code from the Gateway to Global Aging Data platform for harmonizing life history data from SHARELIFE (Wahrendorf et al., 2023). SHARELIFE provides longitudinal information on several aspects of the life course, including work and family. It is a retrospective survey of older people, potentially susceptible to memory bias. However, studies comparing the retrospective SHARELIFE data with the regular SHARE data found negligible inconsistencies between responses. For example, Garrouste and Paccagnella (2011) showed that SHARELIFE respondents remembered their past employment, partnership and parenthood events fairly well, with < 10% recall error. Havari and Mazzonna (2015) further validated the accuracy of other retrospective information in SHARELIFE, such as childhood health and socio-economic status. SHARELIFE offers reasonably accurate retrospective information, as it follows a life history calendar technique (Schröder, 2011), which helps respondents recall past events better.

Using probabilistic sampling and computer-aided face-to-face personal interviewing, SHARELIFE collects data from people aged 50+ across Europe and Israel. The first SHARELIFE was conducted in 2008–2009 in 13 countries and targeted individuals born before 1957. The second SHARELIFE was fielded in 2017 in 28 countries and targeted people who did not participate in the first round and were born before 1967. Both rounds also involved the respondents' partners living in the same household (if applicable), irrespective of their age. The respondents are representative of the European population aged 50+ at the moment of the interview and have their residence in the respective country. Here, we describe the past life histories of people. Our sample may thus not necessarily be representative of the older population residing in a given country during the period our analysis covers.

We applied some selection criteria. First, we removed respondents from Israel (*n* = 2,131) because we focus on European welfare regimes. Second, we excluded persons younger than 50 (*n* = 1,413) as we aim to reconstruct full work-family trajectories before age 50 with equal sequence length. We do not look at work-family trajectories after age 50 because this would result in unequal and incomplete life courses for many respondents. Third, we omitted people who retired before age 50 (*n* = 2,092) to capture the trajectories of those who were not yet withdrawn from the labor market, ensuring that we do not include a selective group. Fourth, we dropped participants with missing information in their work or family histories (*n* = 7,370; after filling in missing work states up to 5 years), remaining with a sample of 78,698 individuals in 28 European countries. Finally, for the multivariate analysis, we deleted cases with missing values on the predictors (*n* = 1,186). Respondents in our analytical sample (*N* = 77,512) are aged 50–104 (*M* = 66.65, *SD* = 9.62) and born between 1908 and 1967.

### 3.2 Measurements

#### 3.2.1 Work trajectories

We defined work trajectories with seven mutually exclusive states combining annual information from age 15 to 49 on paid work, unpaid work and not working: (1) full-time employment, (2) part-time employment, (3) self-employment, (4) unemployment, (5) disability, (6) non-employment, and (7) missing. To code these states, we used the start and end dates of job spells and the respondent's self-reported employment status (i.e., full-time, part-time, or self-employment) based on a categorical variable. For the periods in which no paid work was reported, gaps refer to either unemployment, disability or non-employment. Unemployment covers both searching and not searching for a job. Disability refers to the inability to work because of ill health. Non-employment includes full-time education, home/family work, voluntary/community work and other events (e.g., military service, traveling). Lastly, we included gaps of up to 5 years for which no information was available as missing. We chose 5 years as the upper limit because it enabled us to retain an optimal amount of data. Further details on the construction of work trajectories are given in the Supplementary material.

#### 3.2.2 Family trajectories

Family trajectories are measured by six mutually exclusive states integrating annual information from age 15 to 49 on partnership and parenthood: (1) single, no children, (2) single, children, (3) partnered, no children, (4) partnered, children, (5) unpartnered, no children, and (6) unpartnered, children. We again used each episode's start and end date. Partnership states are based on when a respondent started living with a partner and/or when they stopped living together. Single implies that a person is not in a married/cohabiting partnership (including those never married/cohabited), though they can be in a living-apart-together relationship. Partnered means that a person is in a married/cohabiting partnership (97% of the partnered states concern marriage). Unpartnered reflects whether partners broke up, a partner died or other dissolution events (e.g., moving to a nursing home; 83% of the unpartnered states concern divorce). After creating partnership states, we merged them with whether or not the respondent had a living child. To code parenthood, we used the child's birth year for biological children and the adoption year for adopted children. We did not differentiate between the number of children because that increases the number of family states for each partnership state, which would then run into computational memory issues during the cluster analysis, as the analysis has a limit on the number of sequences. Different from the work trajectories, respondents with at least one missing family state were excluded from the analysis. The rationale for this exclusion and further details on the construction of family trajectories can be found in the Supplementary material.

#### 3.2.3 Predictors

Table 1 provides descriptive statistics for the predictor variables.

**Table 1:** Descriptive statistics of the predictor variables (*N* = 77,512).

	**%**
**Gender**	
Female	55.97
Male	44.03
**Educational level**	
Low educated	37.68
Moderate educated	40.94
High educated	21.38
**Birth cohort**	
Younger cohort	59.76
Older cohort	40.24
**Welfare regime**	
***Southern European***	***21.10***
Cyprus	1.41
Greece	4.48
Italy	6.37
Malta	1.47
Portugal	1.30
Spain	6.09
***Social-democratic***	***11.35***
Denmark	4.65
Finland	2.20
Sweden	4.51
***Eastern European***	***27.20***
Bulgaria	2.23
Croatia	2.57
Czechia	5.74
Hungary	1.74
Poland	6.10
Romania	2.25
Slovakia	2.45
Slovenia	4.11
***Conservative***	***29.52***
Austria	4.19
Belgium	7.09
France	5.17
Germany	5.67
Luxembourg	1.45
Switzerland	3.42
The Netherlands	2.54
***Liberal (Ireland)***	***0.83***
***Baltic***	***9.98***
Estonia	5.75
Latvia	1.86
Lithuania	2.38

The bold italic values refer to the total of values under the given category.

##### 3.2.3.1 Gender

Gender is a binary variable, coded male or female.

##### 3.2.3.2 Educational level

Educational level is the highest degree achieved based on the International Standard Classification of Education 1997 (ISCED 97). We condensed the seven ISCED 97 codes into three categories: low educated (codes 0 = pre-primary education, 1 = primary education, 2 = lower secondary education), moderate educated (codes 3 = secondary education, 4 = post-secondary non-tertiary education) and high educated (codes 5 = first stage of tertiary education, 6 = second stage of tertiary education).

##### 3.2.3.3 Birth cohort

Birth cohort is a categorical variable, classifying people into two broader groups according to their birth year. We coded birth years until 1945 as older cohorts and birth years after 1945 as younger cohorts to distinguish between pre- and post-war cohorts. This is because WWII is considered to be a turning point for economic expansion, modernization and industrialization, which strongly influenced people's life course (Crystal, 2018). Yet, we also tried birth cohort as a linear variable and divided birth years into more than two groups. For example, the results based on four groups of birth cohorts (before 1940, 1940–1945, 1946–1950, and after 1950) can be seen in the Supplementary material. Because these alternative specifications did not lead to different conclusions and birth cohorts based on multiple groups showed similar patterns of results, we preferred the simpler, binary measurement.

##### 3.2.3.4 Welfare regime

We grouped countries into six welfare regime types based on Esping-Andersen (1990, 1999) and others (e.g., Ferrera, 1996), which is a grouping that also largely overlaps with typologies suggested by feminist scholars, including Leitner (2003) and Bambra (2004), and has been used in previous studies, like Uccheddu et al. (2022). Southern European regimes included Cyprus, Greece, Italy, Malta, Portugal and Spain. Social-democratic regimes involved Denmark, Finland and Sweden. Eastern European regimes covered Bulgaria, Croatia, Czechia, Hungary, Poland, Romania, Slovakia and Slovenia. Baltic regimes involved Estonia, Latvia and Lithuania. Conservative regimes contained Austria, Belgium, France, Germany, Luxembourg, Switzerland and the Netherlands. We listed Ireland under the liberal regime, being the only country in SHARELIFE data falling into this welfare regime type. When we examined the prevalence of work-family trajectories by individual countries rather than welfare regime types, we arrived at similar conclusions (see Table A3).

### 3.3 Analytic strategy

#### 3.3.1 Trajectory construction

We applied multichannel sequence and cluster analysis using the TraMineR (Gabadinho et al., 2011) and WeightedCluster (Studer, 2013) packages in R to construct early-to-midlife work-family trajectories. Multichannel sequence analysis is an extension of sequence analysis, which is one of the most established holistic approaches to constructing life course typologies from longitudinal categorical data (Aisenbrey and Fasang, 2010; Gauthier et al., 2010). Therefore, it provides an ideal tool for the methodological implementation of the theoretical notion of “trajectory” by allowing us to identify (and visualize) sequences of different states and transitions that are dissimilar from one another.

We calculated dissimilarities between individual sequences with the optimal matching metric, which measures the extent to which each pair of individual sequences is dissimilar based on how costly it is for one sequence to turn into another. For this, we needed to specify two types of costs: substitution costs and insertion/deletion (indel) costs. Substitution costs are the costs of replacing a state in a sequence with another state. Indel costs are the costs of inserting or deleting a state from a sequence. We used a user-defined cost matrix (see Tables A1.1, A1.2) instead of following the common procedure in which all costs are set equal. This enabled us to make theoretical choices in the matching process by taking into account that some transitions can be considered more costly than others. For example, the transition from full-time employment to unemployment can be regarded as more costly than the transition from full-time to part-time employment.

After obtaining a matrix of pairwise dissimilarities, we subjected it to Ward hierarchical clustering to assess whether similar sequences can be grouped into homogeneous groups. To determine the appropriate number of clusters, we reviewed the content of clusters and inspected multiple statistical cut-off criteria, including the Average Silhouette Width (ASW), Hubert's Gamma Somers' D (HGSD) and the Point Biserial Correlation (PBC)^2^. Because this cluster analysis has a limit on the number of unique sequences (around 43,000), we aggregated work-family sequences and used unique sequences in the analysis. This is why we also had to limit the age range to 15–49 and combine some employment, partnership and parenthood states. Finally, since we aimed to provide “average” life courses for a broader group, we did not calculate work-family trajectories separately for men and women (cf. Komp-Leukkunen, 2019; Schmitz et al., 2023). However, multichannel sequence and cluster analysis is powerful enough to capture gender differences (Aisenbrey and Fasang, 2010). If a cluster exists among only men or only women, it will emerge as a cluster in the analysis based on the combined sample of men and women^3^. The code for reproducing the work-family trajectories is freely available at Open Science Framework (OSF): https://osf.io/njqpd/.

#### 3.3.2 Trajectory membership

We examined trajectory membership by gender, education, birth cohort and welfare regime using average marginal effects (AMEs). We used AMEs because they make the interpretation of the results more intuitive. AMEs show the average change in the probability of being observed in a trajectory given the change in an independent variable for each observation in the sample, holding all other predictors constant. We calculated AMEs through Stata's margins command after running multinomial logistic regression models. We conducted multinomial logistic regression analyses on the (1) total sample, (2) male sample, and (3) female sample. Gender differences in the role of education, birth cohort and welfare regime were tested by performing a binomial logistic regression analysis for each trajectory and inspecting the significance of the logit of the gender interaction term, as currently there is no agreed-upon way of correctly computing the AMEs of interaction terms in Stata for multinomial models. In all analyses, we took into account that observations within countries are not independent of one another by estimating robust standard errors clustered at the country level^4^.

## 4 Results

### 4.1 Work-family trajectories

The statistical cut-off criteria suggested two optimal numbers of clusters. The ASW (0.46) suggested three clusters, but HGSD (0.89) and the PBC (0.73) suggested six clusters (see Figure A1). We adopted the six-cluster solution for two reasons. First, the six-cluster solution was more informative and diverse, providing richer insights into the multiplicity of work-family configurations over the earlier life course. Second, the ASW value for the six-cluster solution (0.40) was above the acceptable level of 0.25.

**Figure 2:**
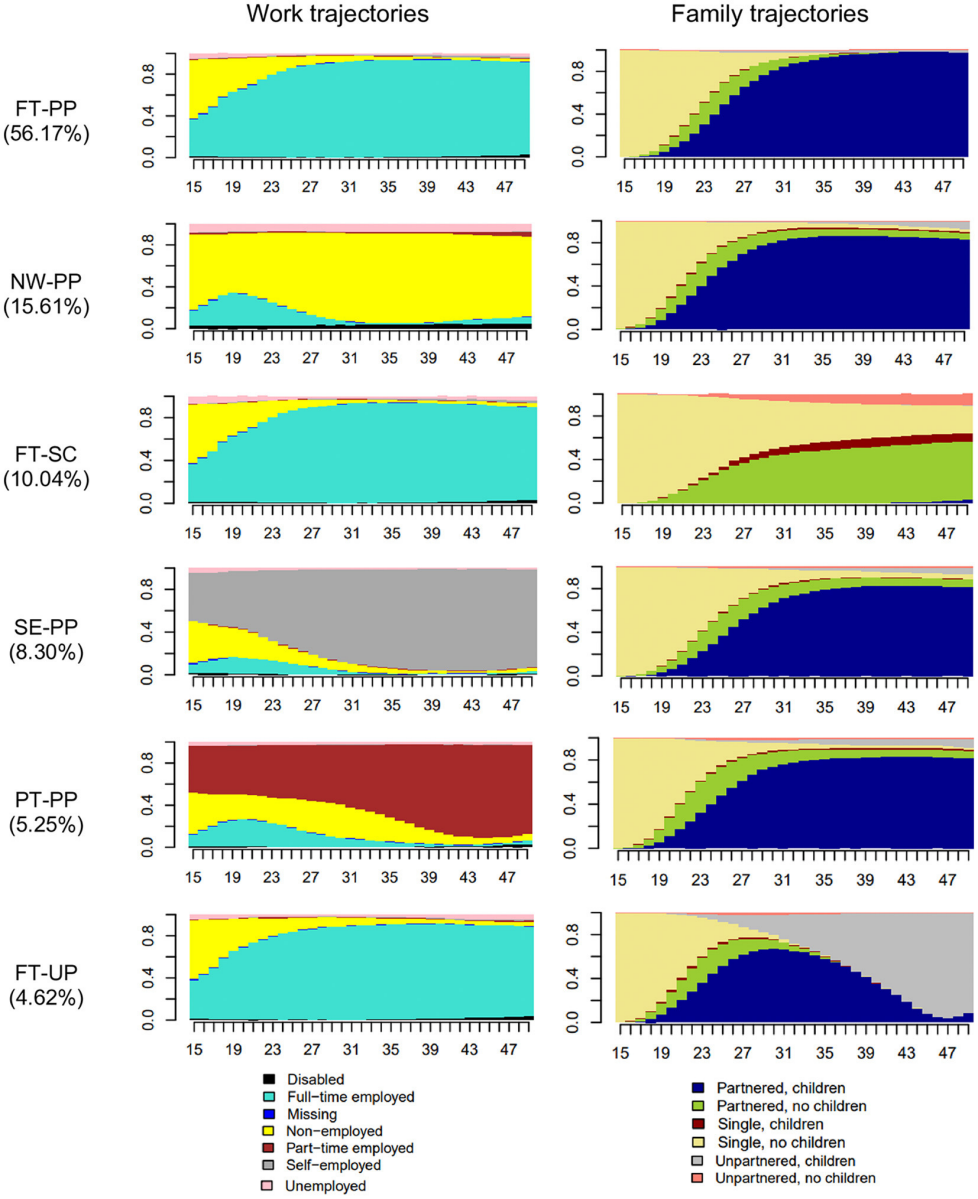
State distribution plots of work-family trajectories from the age of 15–49. The x-axis indicates age (from 15 to 49). The y-axis indicates the proportion (0–1) of individuals in a given work and family state. FT-PP, full-time worker, partnered parent; NW-PP, non-worker, partnered parent; FT-SC, full-time worker, single/childless couple; SE-PP, self-employed, partnered parent. PT-PP, part-time worker, partnered parent; FT-UP, full-time worker, unpartnered parent.

Figure 2 displays the state distribution plots of the six clusters ordered from the highest to lowest prevalence. Work trajectories are shown on the left side and the corresponding family trajectories are on the right. The plots reflect the proportion of individuals in a given work and family state at each age from 15 to 49. The labels we assigned to the clusters are based on their dominant states. Tables A2, A3 provide descriptive information on the clusters' social composition.

The first cluster, *full-time worker, partnered parent*, is the largest. Therefore, it represents the standard work-family trajectory in early to midlife in our sample. It is characterized by continuous full-time employment and having a partner with children. People in this cluster spend on average 28.9 years (*SD* = 5.9) in full-time employment and 23.4 years (*SD* = 5.0) in a partnership with children, with full-time employment prevailing after the mid-20s and partnership with parenthood after the 30 s.

The other clusters are less prevalent and thus non-standard work-family trajectories in our sample over their earlier life course. The second one is a female trajectory, made up of ~95% of women who are a *non-worker, partnered parent*. Non-employment is the dominant work state, with an average of 26.7 (*SD* = 10.5) years. There is full-time employment before age 30, but non-employed takes over after marriage/childbirth.

The third cluster, *full-time worker, childless single/couple*, resembles the first cluster in terms of the work trajectory, as people who follow this trajectory are in full-time employment for an average of 28.9 (*SD* = 6.2) years. The difference is that people in this cluster do not form a traditional family. They either stay single (*M* = 18.1, *SD* = 11.6) or they do not have a child when they have a partner (*M* = 12.9, *SD* = 11.5). After age 30, some unpartnered childless people also appear in this cluster.

The fourth cluster, *self-employed, partnered parent*, is a male trajectory, with two-thirds of its members being men. What sets this cluster apart from the previous ones is the work trajectory. Individuals in this cluster are self-employed for an average of 28.1 (*SD* = 7.2) years, although we see some full-time employment and non-employment before the 30 s. A nuance in the family trajectory is that having a partner and children concentrates on older ages.

The fifth cluster is again a female trajectory, largely (~90%) comprising women who are a *part-time worker, partnered parent*. Part-time employment (*M* = 22.8, *SD* = 8.8) increases after family formation, but there is full-time employment before age 30 and non-employment until age 40. The family trajectory resembles the second cluster. Nevertheless, in this cluster, the duration of partnership with children is roughly 2 years shorter (*M* = 20.6, *SD* = 9.1) than in the second cluster.

The sixth and smallest cluster, *full-time worker, unpartnered parent*, looks like the first and third clusters with respect to the work trajectory, with an average of 28.3 years (*SD* = 6.5) spent in full-time employment. The distinctive feature of this cluster is the occurrence of a union dissolution event involving children (*M* = 12.8, *SD* = 6.0)—an event becoming common after age 30. Unlike the first and third cluster, this cluster has more women (~71%), making it yet another female trajectory.

### 4.2 Membership in work-family trajectories: main analysis

**Figure 3:**
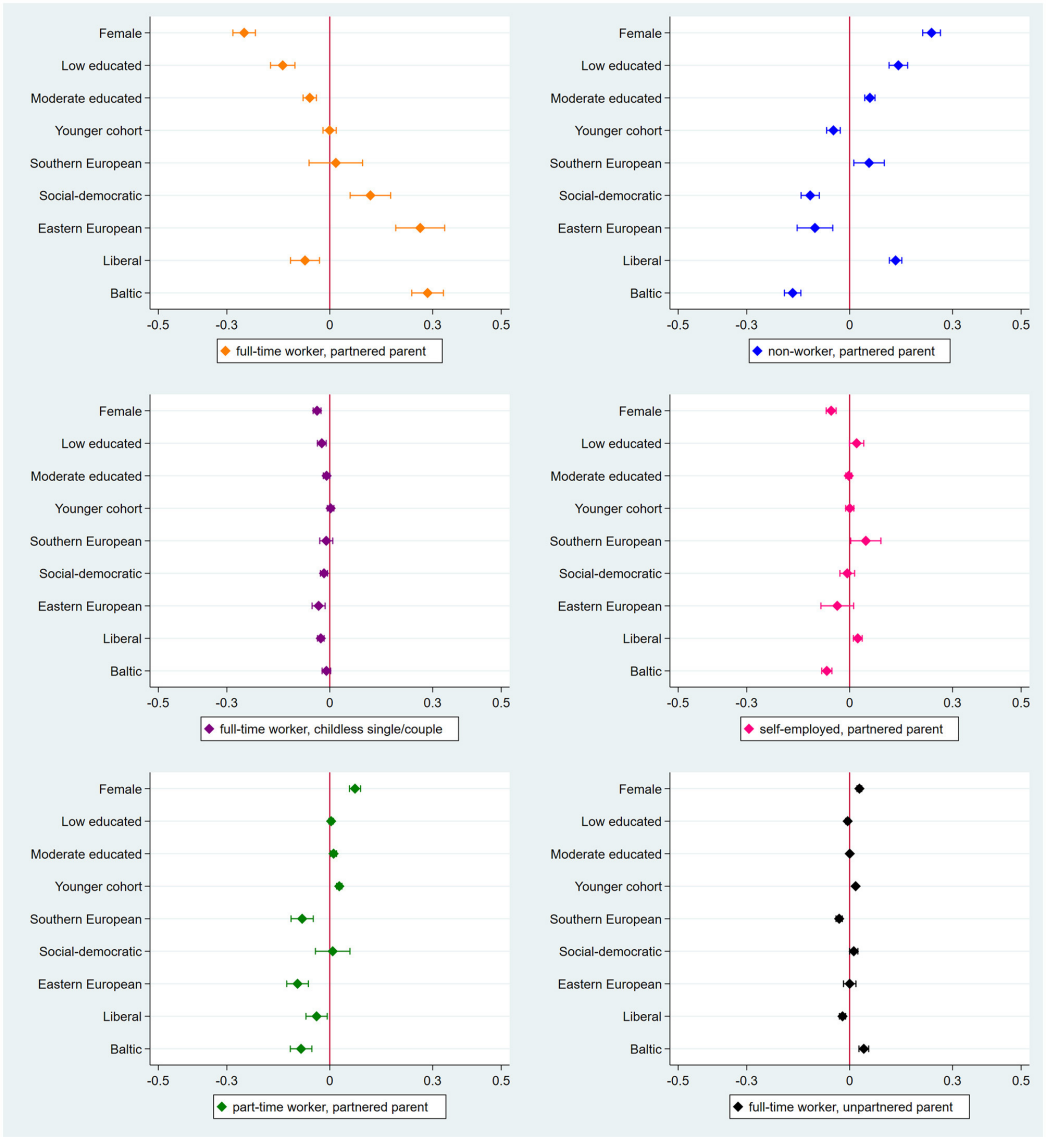
Average marginal effects for membership in work-family trajectories with 95% confidence intervals (*N* = 77,512). Reference categories (male, high educated, older cohort, and conservative welfare regime) are not shown in the figure.

Figure 3 depicts the AMEs of gender, education, birth cohort and welfare regime from the multinomial logistic regression analysis. Table A4 shows all coefficients.

#### 4.2.1 Membership in the standard work-family trajectory

Women (vs. men), people with low and moderate (vs. high) education and people from liberal (vs. conservative) welfare regimes are less likely, whereas people from social-democratic, Eastern European and Baltic (vs. conservative) welfare regimes are more likely to follow the trajectory of full-time worker, partnered parent. The estimated coefficients are especially strong for women (25% points lower likelihood) and for those from Eastern European (26.4% points higher likelihood) and Baltic (28.5% points higher likelihood) welfare regimes. Birth cohort is unrelated to this trajectory.

#### 4.2.2 Membership in non-standard work-family trajectories

##### 4.2.2.1 Gender

Women are more likely than men to be a non-worker and part-time worker, but less likely to be self-employed in conjunction with being a partnered parent. It is worth noting that women display a 23.9% points higher likelihood relative to men to follow the trajectory of non-worker, partnered parent. In terms of the two trajectories that combine full-time employment with a non-standard family arrangement, women exhibit divergent patterns. Compared to men, they are less likely to be in the childless single/couple cluster, yet more likely to be an unpartnered parent (i.e., single mom).

##### 4.2.2.2 Educational level

Low and moderate (vs. high) educated persons are more likely to be a non-worker, partnered parent, which is more prominent for the low educated (14.1% points more likely). Furthermore, the low educated are less likely to be a full-time worker while being a childless single/couple. However, this does not apply to the moderate educated, who are more likely than the high educated to be a part-time worker and partnered parent. We do not observe any educational differences in the likelihood of having a trajectory of self-employed, partnered parent or full-time worker, unpartnered parent.

##### 4.2.2.3 Birth cohort

Younger (vs. older) cohorts are 4.7% points less likely to be a non-worker, partnered parent, yet 2.8 and 1.7% points more likely to be a part-time worker, partnered parent and full-time worker, unpartnered parent, respectively. We find no other cohort differences.

##### 4.2.2.4 Welfare regime

Contrasted with people from conservative regimes, those from Southern European regimes are more likely to be a non-worker and self-employed combined with being a partnered parent. However, they are less likely to be a part-time worker, partnered parent and full-time worker, unpartnered parent. Individuals from social-democratic regimes are 11.5% points less likely than those from conservative regimes to be a non-worker, partnered parent. They are also less likely to be a full-time worker, childless single/couple, but more likely to be a full-time worker, unpartnered parent. Persons from Eastern European regimes are less likely to follow any non-standard trajectory than those from conservative regimes, except for those involving self-employment and basically divorce, as people from Eastern European and conservative regimes do not differ in these trajectories. People from liberal regimes differ from those from conservative regimes in all trajectories. Specifically, they are more likely to be in non-employment (13.4% points) and self-employment trajectories, but less likely to be in single/childless, part-time worker and unpartnered parent trajectories. Individuals from Baltic regimes also differ from their peers in conservative regimes in all trajectories, except for the one including singlehood/childlessness. In particular, persons from Baltic regimes are less likely to be in all non-standard trajectories, except for the unpartnering trajectory, in which they are more likely to be by 4.1% points.

### 4.3 Membership in work-family trajectories: gender differences

**Figure 4:**
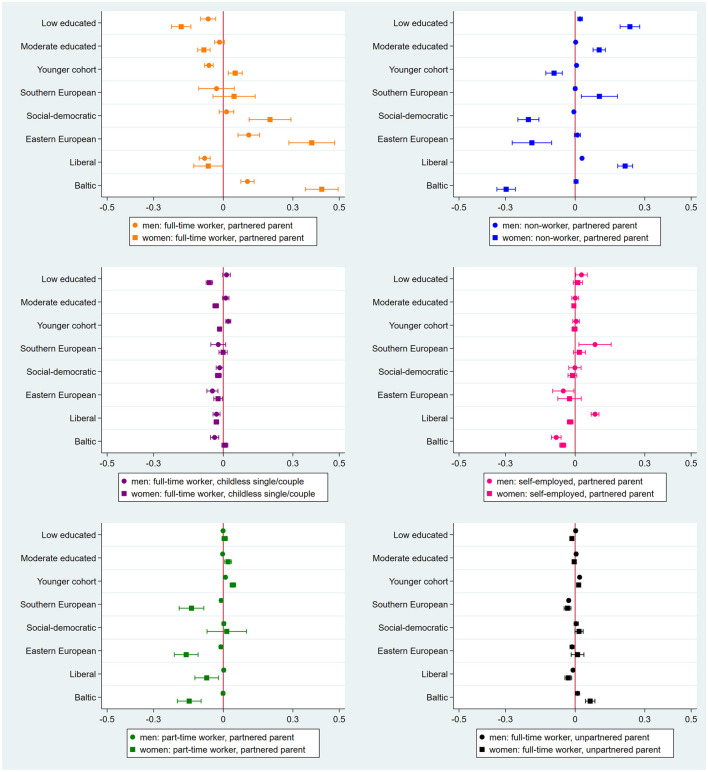
Average marginal effects for membership in work-family trajectories among men (*n* = 34,132) and women (*n* = 43,380) with 95% confidence intervals. Reference categories (high educated, older cohort and conservative welfare regime) are not shown in the figure.

To explore gendered effects, we ran the multinomial logistic regression models separately for men and women (see Figure 4 for the AMEs from these models) and inspected the significance of the logit of the gender interaction terms in logistic regression models (see Table A5).

Starting with the standard work-family trajectory over the earlier life course, we generally find that the differences between educational levels and welfare regimes are in the same direction as the main analysis. What we do see is that the estimated coefficients are usually larger for women. For example, both men and women with low (vs. high) education are less likely to be in the standard trajectory, but this difference is larger for women (18.1% points) than men (6.5% points). Similarly, both men and women from Eastern European and Baltic (vs. conservative) regimes are more likely to be in the standard trajectory, but this difference is again larger for women (38.1% points in Eastern European regime, 42.5% points in Baltic regime) than men (11% points in Eastern European regime, 10.5% points in Baltic regime).

Regarding the non-standard trajectories, we see fewer gender differences for the trajectories involving self-employment and full-time employment. For example, we see that men from liberal regimes are more likely, whereas women from liberal regimes are less likely to be self-employed in conjunction with having a partner and child. As another example, we find that both men and women from Baltic regimes are more likely to unpartnered in conjunction with working full-time, while this effect is again stronger for women (6.5% points) than men (1% points). However, we find the most relevant gender differences for the trajectories of non-worker and part-time worker partnered parents. For instance, women with low (vs. high) education are 23.6% points more likely to be a non-worker, partnered parent, whereas this difference is rather small among men from low and high educated backgrounds (2.1% points). Furthermore, women from social-democratic, Eastern European and Baltic (vs. conservative) regimes are over 20% points less likely to be a non-worker, partnered parent. Additionally, women from Southern, Eastern European and Baltic (vs. conservative) regimes are over 13% points less likely to be a part-time worker, partnered parent. Yet, these effects do not really exist among their male counterparts.

The gendered analysis of cohort differences presents a contrasting yet informative pattern of results. First, we observe that some coefficients are in opposite directions for men and women. Compared to their older counterparts, younger cohorts of women are more likely, whereas younger cohorts of men are less likely to be in the standard trajectory. Also, younger male cohorts are more likely to be a non-worker, partnered parent and full-time worker, childless single/couple than older male cohorts. In contrast, younger female cohorts are less likely to follow these trajectories than older female cohorts. Second, we find no differences between men and women in the role of cohort in being a self-employed and part-time worker with being a partnered parent. Lastly, we see a slightly larger cohort difference among men in the full-time worker, unpartnered parent trajectory relative to women.

## 5 Discussion

In the present study, we aimed to overcome the limited scope and generalizability of previous studies by providing the most comprehensive empirical description of early-to-midlife work-family trajectories and their social stratification to date. To this end, we used rich retrospective life history data of almost 80,000 respondents from 28 European countries and observed work-family trajectories from age 15 to 49. This complementary approach, which enabled us to identify common earlier life courses that apply to a wider population, proved fruitful and led to insightful conclusions.

Our first conclusion is that it is possible to distinguish early-life work-family trajectories across such a high number of countries and individuals despite their many differences. To distinguish work-family trajectories, we applied multichannel sequence and cluster analysis. This is a holistic method to empirically study the theoretical concept of trajectory. This data-driven explorative approach revealed six distinct work-family trajectories over the earlier life course. The most prevalent trajectory in Europe can be considered a standard or common one, characterized by continuous full-time employment in the work domain and having a stable relationship with children in the family domain. The other five are non-standard, again statistically speaking, in either the work or family domain. Three of them combine non-standard work trajectories with a standard family trajectory; the remaining two combine a standard work trajectory with non-standard family trajectories. These findings confirm our initial presumption that non-standard work-family trajectories exist next to a standard one. It also confirms the added value of our comprehensive approach. We included more states defining the work and family sequences that also covered a larger part of the earlier life course. Therefore, we could capture some trends that prior research using similar data could not identify. For example, Lesnard et al. (2016) and Schwanitz (2017) studied trajectories until age 35 and omitted several states in both the work (e.g., full-time and part-time employment) and family (e.g., divorce and widowhood) sequences. Yet, with our design, we uncovered a work-family trajectory that was characterized by union dissolution (mostly divorce) after age 30 while working full-time.

Our second conclusion is that early-to-midlife work-family trajectories are strongly socially stratified by gender, education, birth cohort and welfare regime. Only a few previous studies included these factors together, albeit with a smaller sample size and a smaller number of countries and states. Moreover, they looked at work-family trajectories at younger ages only (e.g., age 18–34; Schwanitz, 2017). Our broader approach helped us better evaluate how earlier-life work-family trajectories are stratified. For example, we found that stratification by gender is widespread and entrenched across societies despite policy efforts to support the dual-earner/dual-carer model. Hinting at the persistence of the male breadwinner and female homemaker norm, women have more restricted access to the standard work-family trajectory over the earlier life course, but this is changing for younger female cohorts. Women often do not work or work part-time after they get married or have children. When they work full-time, they tend to be divorced or widowed. These work-family constellations at younger ages can hinder their life satisfaction, financial wellbeing or career mobility at older ages, widening social inequalities (Damman et al., 2015; Madero-Cabib and Fasang, 2016; Ponomarenko, 2016; Visser et al., 2018). Lower educated persons are alike. Reflecting their limited human capital, the lower educated are underrepresented in the standard work-family trajectory. Instead, they are non-employed or part-time employed for large parts of their early and midlife while having a nuclear family. Such a pattern may, for instance, force them to postpone retirement, as they would have fewer pension savings, which could increase inequalities in extending working lives (Visser et al., 2016b).

Our third conclusion is that not only early-to-midlife work-family trajectories are gendered, but also the relations between these trajectories and education, birth cohort and welfare regime. This is relevant because it helps clarify the gendered nature of stratification by other social attributes, which has been previously acknowledged but not empirically well-examined (Becker, 1981; Sainsbury, 1999; Bukodi et al., 2012). We did so, uncovering divergent effects of education, historical time and institutional context on men's and women's lives. For instance, compared to lower educated men, lower educated women have an even lower likelihood of following work-family trajectories that involve full-time employment (with and without standard family formation) in the earlier life course. In contrast, lower educated women (vs. men) display an even higher likelihood of following work trajectories dominated by non-employment and part-time employment along with a traditional family in the earlier life course. Thus, education seems to have unequal returns for men and women in the work-family domain from early to midlife (Berrington and Pattaro, 2014; Jalovaara and Fasang, 2015).

The results for birth cohort were counterintuitive at first, but the gendered analysis shed light on them. The standard trajectory did not seem to have become less common over time and birth cohort was not associated with a family trajectory characterized by being single or a childless couple, which contradicts the life course destandardization hypothesis (Brückner and Mayer, 2005). Yet, by splitting the analysis for men and women, we found support for this hypothesis for men, as they have become less likely to follow the standard trajectory. Interestingly, we found the exact opposite in women, which can be attributed to their emancipation and increased educational attainment, seemingly rejecting the destandardization hypothesis. We argue that the destandardization hypothesis needs to be refined from a gender perspective so that we can accurately interpret how earlier-life work-family trajectories change over time for men and women. What was standard in the past for men is not the same as for women, and what can be considered standard for older cohorts of women is partly changing into the standard trajectory of mostly older male cohorts. Without examining cohort by gender, we would have missed this discrepancy, drawing misleading conclusions. By going beyond prior work (e.g., Uccheddu et al., 2022), we were able to draw a more accurate picture of gendered life courses. Yet, we did not look at cohort differences by country, which can be done in future research, considering that work and family life courses may have taken divergent pathways across countries over time (e.g., Perelli-Harris and Lyons-Amos, 2016; Van Winkle and Fasang, 2017).

The way welfare regimes shape early-to-midlife work-family trajectories is also gendered. We theorized that people from social-democratic, Eastern European and Baltic regimes would be more in the standard trajectory but less in non-standard ones due to high decommodification, low familialism and high defamilialism. The results largely corroborated this theorizing, with clear gender differences. We showed that especially women living in these countries are more likely to follow a standard work-family trajectory and less likely to follow non-standard work trajectories dominated by non-employment or part-time employment from early to midlife. This conveys two messages. First, welfare regimes are more relevant for women and shape earlier work-family trajectories in gendered ways because of their gendered policies (Aisenbrey and Fasang, 2017). Second, welfare regimes differ in their equalizing potential (Sainsbury, 1999). Consistent with their gender-equal practices, it seems that social-democratic, Eastern European and Baltic regimes enable women to actively combine work and family (Van Winkle and Fasang, 2021). Although this confirms the historical context in which our sample formed their life, it is a remaining question if these results hold for Eastern European women born after the fall of communism, which may have diversified life courses (Lesnard et al., 2016). For instance, we saw that women from Baltic regimes are more likely to be unpartnered with full-time work than women in conservative regimes, which deserves further research and supports our choice to separate Baltic regimes from Eastern European regimes, as this difference was found to be unique to Baltic regimes. Future research is suggested to disentangle the mechanisms that underlie this discrepancy, with a focus on institutional differences between Eastern European countries in the transition from communism to democracy.

The results for Southern European regimes were mixed compared to previous studies. On the one hand, we found that non-employment and self-employment with a standard family are more common in Southern European regimes, supporting past evidence (Torrini, 2005; Ponomarenko, 2016). Extending past evidence, we showed that the prevalence of non-employment in these regimes is driven by women, and self-employment is driven by men. On the other hand, we found that part-time work with a traditional family and full-time work with a dissolved family are less common in Southern European regimes. Although inconsistent with past evidence, these findings make sense because, relative to conservative regimes, part-time work is less established and union dissolution is less normative in Southern European regimes (Bosch, 2004; Van Winkle, 2018).

Regarding liberal regimes, the results were mostly in line with previous studies. For instance, similar to Komp-Leukkunen (2019) and Schmitz et al. (2023), we also found that people from liberal regimes are less likely to be in the standard trajectory and more likely to be in non-employment and self-employment trajectories, as expected. However, we extended the previous evidence by capturing more nuanced family dynamics. For example, we captured trajectories characterized by singlehood, childlessness and union dissolution and showed that people from liberal regimes are less likely to be in these trajectories, especially if they are female. Overall, we could draw more substantial conclusions about how welfare regimes shape gendered work-family trajectories in the early life course, as we included more welfare regimes covering more countries with larger samples and broader work-family states.

Nevertheless, some limitations should be kept in mind while interpreting our findings. First, we shed light on the social stratification of early-to-midlife work-family trajectories; however, we did not illuminate whether people follow a given trajectory willingly or reluctantly. The reason is that SHARELIFE, like most other life surveys, does not provide retrospective information on the voluntariness of work and family transitions. Considering that one's vulnerability may intensify if one takes non-standard paths not because of individual preferences but because they are forced to, future research should investigate the conditions under which people follow standard and non-standard work-family trajectories voluntarily or involuntarily.

Second, although we captured the most salient events in people's earlier work and family life and covered more work and family states than prior studies, there is still a lot we could not observe, which makes some of our categorizations seem like rather unspecific. For example, we did not distinguish between marriage and cohabitation or divorce and widowhood. We also did not consider the age of children, the number of children or whether children live in the same household. Moreover, families are becoming increasingly complex because of re-partnering and the presence of stepchildren. Similarly, we could not take into account some aspects of work, such as supervisory status and contract type (e.g., temporary or permanent). Including these dimensions could have enriched our understanding of early-life work-family trajectories. However, it would have complicated the multichannel sequence and cluster analysis (due to computer memory issues) and the results would have been harder to interpret. Relatedly, we did not include care responsibilities in the family trajectories because SHARELIFE does not involve data regarding caring histories. Future research is suggested to include care trajectories, considering that care policies across countries may influence gender inequality in paid and unpaid work in different ways (Leitner, 2010; Saraceno and Keck, 2011).

Third, we followed the well-known welfare regime approach to characterize cross-national variation in early-to-midlife work-family trajectories, which helped us overcome the complexity of analyzing a sizeable number of countries. However, this approach assumes considerable similarities between countries within the same regime. Although we did indeed find considerable similarities between countries within the same regime in the prevalence of the work-family trajectories over the early life course (see Table A3), we may have underestimated cross-national dissimilarities. Instead of a categorical country-level variable, future studies could use variables on which each country scores a unique value (e.g., an index based on work-family policies) to arrive at more substantial conclusions (cf. Van Winkle, 2020).

Despite these limitations, we contributed to the understanding of how early-to-midlife work-family trajectories look like and to what extent they are socially stratified in Europe. We embraced an encompassing approach that covered more individuals and countries than ever before and included four key predictors. Our findings highlighted pronounced disparities in whether people are likely to follow a standard or non-standard work-family trajectory in earlier life based on gender, education, birth cohort and welfare regime. Especially women and the lower educated could be in a disadvantageous position in later life, as they tend to be in non-standard work-family trajectories in earlier life that often create inequalities. Against the background of aging societies, precarious employment and increasing family complexity, future studies are invited to examine whether non-standard work-family trajectories have adverse effects on later-life outcomes, such as retirement and wellbeing, and to what extent government policies can protect vulnerable social groups against these risks. To complement our invitation, we make our code producing the trajectory data publicly available, with the hope that it facilitates future research.

## Data availability statement

In accordance with the SHARE conditions of use, we are not allowed to share data. The code for reproducing the dataset generated/analyzed for this study can be found at OSF: https://osf.io/njqpd/. Requests to access these datasets should be directed to MF, mustafa.firat@ru.nl.

## Ethics statement

The studies involving humans were approved by Ethics Committee of the University of Mannheim and the Ethics Council of the Max Planck Society. The studies were conducted in accordance with the local legislation and institutional requirements. The participants provided their written informed consent to participate in this study.

## Author contributions

MF, MV, and GK developed the idea. MF prepared the data, conducted the analysis, and wrote the original draft. MV and GK reviewed and edited the manuscript, supervised the project, and acquired the funding. All authors contributed actively to the article and approved the submitted version.

## Footnotes

^1^At least two drawbacks are worth noting. First, work-family trajectories are more nuanced than a mere standard vs. non-standard distinction. For example, self-employment denotes a non-standard work arrangement. Yet, there are emerging forms of self-employment (e.g., gig or platform economy workers) that can be regarded as more non-standard than the traditional self-employed (e.g., business or shop owners). While recognizing these nuances, we do not explore them because of data limitations and conceptual simplicity. Second, standard work-family trajectories traditionally refer to a heterosexual-male normative life course of continuous full-time employment with having a stable relationship and children. Although our results show that such a trajectory is the most common one among women as well, we want to emphasize that our distinction regarding standard vs. non-standard trajectories does not refer to normative life courses.

^2^We tried two alternative cost specifications. One included the common procedure of setting substitution costs as 2 and indel costs as 1. The other included Partitioning Around Medoids (PAM). Both analyses delivered similar work-family trajectories to the ones we report in the results, with minor differences. We decided to report the current solution because it (a) provided a richer picture, covering all (and more than) the work-family patterns observed in the alternative solutions, (b) yielded better or comparable clustering quality indices, and (c) demonstrated slightly better predictive validity. We also tried the Dynamic Hamming Distance (DHD) metric. However, due to computer memory issues, we could not obtain a reliable cluster solution.

^3^As a robustness check, we ran the multichannel sequence and cluster analysis separately for men and women. We found similar clusters to the ones reported in the results. The major difference was that the trajectories of non-employment and part-time employment emerged only among women. This corroborates the power of our analytic strategy, suggesting that splitting the multichannel sequence and cluster analysis for men and women does not matter for capturing gender differences. The reason is that we already observe these gender differences in the results, finding that the trajectories of non-employment and part-time employment are female trajectories, with more than 90% of the members being female.

^4^We also estimated two-level multilevel models (i.e., individuals nested within countries). However, we decided to report the models using robust standard errors. We made this decision for two reasons. First, we could not obtain average marginal effects from the multilevel models, making the interpretation of the findings less intuitive. Second, we compared the logit coefficients from both analyses, with the results being similar. In fact, the models with robust standard errors were often more conservative than the multilevel models. The results reporting the logit coefficients from both analyses can be found in the Supplementary material.
