# Supplementary material for: Work-family trajectories across Europe: differences between social groups and welfare regimes
Source: Front Sociol. 2023 Nov 30;8:1100700. doi: 10.3389/fsoc.2023.1100700 (PMC10720591; doi:10.3389/fsoc.2023.1100700)
Supplement: Supplementary file 1 [file Data_Sheet_1.pdf]

Firat M, Visser M and Kraaykamp G (2023) Work-family trajectories across Europe: Differences between social groups and welfare regimes. *Front. Sociol.* 8:1100700. doi: 10.3389/fsoc.2023.1100700

Supplementary Material

1 Work Trajectories

When a respondent simultaneously declared paid work, unpaid work and not working, we prioritized unpaid work and not working. In both waves of SHARELIFE, respondents indicated whether a job spell was always full-time, always part-time or involved (multiple) changes between full-time and part-time work. We used this information to distinguish working hours. In the first wave, respondents could report changes in working hours within the same job spell. Therefore, we could capture all changes within the same job spell in the first wave. However, in the second wave, this option was not offered. Therefore, we did the following for the second wave: if a respondent reported a change from part-time to full-time, we considered this spell part-time. If they reported a change from full-time to part-time, we considered this spell full-time. That is, we relied on the starting status. If multiple changes in working hours were reported, we did not code this spell (left it missing) because we had no clue about the starting status.

Before creating work states, we removed participants who did not report start or end dates, employment type and working hours of job spells. This approach prevented us from creating inaccurate sequences. After creating work states, we filled in some missing states in two steps. First, we filled in missing states corresponding to the years between 1939 and 1945 with a Second World War (WWII) gap. However, because the number of WWII gaps was low (n = 194 respondents, 480 person-years), we excluded respondents coded with this gap. Second, we created and included a missing state in the analysis for missing information up to 5 years (n = 2,854 respondents, 6,442 person-years). It is unlikely that this strategy has affected our findings because the number of missing states was small, accounting for only 0.23% of work states across all time points and respondents.

2 Family Trajectories

We combined marriage and cohabitation as partnered because cohabitation was uncommon for our study cohorts. Moreover, distinguishing between marriage and cohabitation would increase the number of states with the addition of parenthood, which could lead to unclear grouping of sequences in the cluster analysis. This is why we did not distinguish between divorce and widowhood either.

Before constructing family states, we removed respondents who did not report partnering episodes' start and/or end dates. This, again, protected us from forming inaccurate sequences. After constructing the family states, we deleted people with a missing state. Unlike the work trajectories, we did not use a missing state in family trajectories for two reasons. First, missingness in the family was even lower (total n = 1,736, 1.89% of the original sample), making it less costly to drop them. Second, it would have meant two additional states (‘missing, children’ and ‘missing, no children’, or an additional state of ‘missing’ that incorporates children and no children), which would increase the number of sequences and could lead to computer memory issues during the analysis.

We also addressed multiple partnering episodes within the same year. When an episode's start and ending happened in the same year, we moved the ending one year forward. If the old episode's ending and the new one's start occurred in the same year, we moved the new episode one year forward. We did not apply this strategy when such situations happened at the end of the trajectory, in which case the previous state was held.

Table S1

Multinomial logistic regression analysis of work-family trajectories (*N* = 77,512), multinomial logit coefficients (*B*) with robust standard errors (*RSE*) clustered at the country level

	non-worker, partnered parent *vs.* full-time worker, partnered parent		full-time worker, childless single/couple *vs.* full-time worker, partnered parent		self-employed, partnered parent *vs.* full-time worker, partnered parent		part-time worker, partnered parent *vs.* full-time worker, partnered parent		full-time worker, unpartnered parent *vs.* full-time worker, partnered parent	
	B	*RSE*	*B*	*RSE*	*B*	*RSE*	*B*	*RSE*	*B*	*RSE*
**Gender**										
Female	3.341***	0.227	0.147	0.076	-0.028	0.087	2.549***	0.185	1.089***	0.055
Male	Ref.	Ref.	Ref.	Ref.	Ref.	Ref.	Ref.	Ref.	Ref.	Ref.
**Educational level**										
Low educated	1.711***	0.110	0.055	0.068	0.538***	0.126	0.570***	0.084	0.172*	0.068
Moderate educated	0.865***	0.085	0.030	0.052	0.084	0.072	0.424***	0.101	0.132**	0.048
High educated	Ref.	Ref.	Ref.	Ref.	Ref.	Ref.	Ref.	Ref.	Ref.	Ref.
**Birth cohort**										
Younger cohort	-0.388***	0.100	0.022	0.051	-0.014	0.090	0.557***	0.094	0.402***	0.055
Older cohort	Ref.	Ref.	Ref.	Ref.	Ref.	Ref.	Ref.	Ref.	Ref.	Ref.
**Welfare regime**										
Southern European	0.205	0.229	-0.136	0.143	0.396	0.243	-1.542***	0.265	-1.115***	0.140
Social-democratic	-1.356***	0.221	-0.404***	0.086	-0.330*	0.165	-0.295	0.321	-0.077	0.086
Eastern European	-1.517***	0.357	-0.814***	0.130	-1.000*	0.490	-2.937***	0.312	-0.588***	0.151
Liberal	0.961***	0.122	-0.071	0.082	0.425***	0.100	-0.124	0.215	-0.334***	0.082
Baltic	-2.794***	0.329	-0.605***	0.097	-1.910***	0.201	-2.342***	0.258	-0.002	0.108
Conservative	Ref.	Ref.	Ref.	Ref.	Ref.	Ref.	Ref.	Ref.	Ref.	Ref.
**Intercept**	-4.135***	0.191	-1.460***	0.099	-1.830***	0.108	-3.900***	0.259	-3.185***	0.101

*$p < 0.05$, **$p < 0.01$, ***$p < 0.001$ (two-tailed).

Table S2

Multinomial logistic regression analysis of work-family trajectories among men (*n* = 34,132), multinomial logit coefficients (*B*) with robust standard errors (*RSE*) clustered at the country level

	non-worker, partnered parent *vs.* full-time worker, partnered parent		full-time worker, childless single/couple *vs.* full-time worker, partnered parent		self-employed, partnered parent *vs.* full-time worker, partnered parent		part-time worker, partnered parent *vs.* full-time worker, partnered parent		full-time worker, unpartnered parent *vs.* full-time worker, partnered parent	
	B	*RSE*	*B*	*RSE*	*B*	*RSE*	*B*	*RSE*	*B*	*RSE*
**Educational level**										
Low educated	1.178***	0.215	0.214*	0.085	0.340**	0.118	0.047	0.173	0.172	0.112
Moderate educated	0.223	0.159	0.116	0.069	0.029	0.087	-0.180	0.111	0.168	0.110
High educated	Ref.	Ref.	Ref.	Ref.	Ref.	Ref.	Ref.	Ref.	Ref.	Ref.
**Birth cohort**										
Younger cohort	0.429***	0.117	0.271***	0.054	0.130	0.080	0.966***	0.200	0.800***	0.062
Older cohort	Ref.	Ref.	Ref.	Ref.	Ref.	Ref.	Ref.	Ref.	Ref.	Ref.
**Welfare regime**										
Southern European	0.078	0.232	-0.116	0.156	0.611*	0.245	-0.679**	0.256	-1.214***	0.156
Social-democratic	-0.416	0.240	-0.133	0.068	-0.028	0.139	0.120	0.244	0.094	0.109
Eastern European	0.329	0.265	-0.541***	0.117	-0.751	0.387	-1.038**	0.356	-0.574***	0.147
Liberal	1.189***	0.068	-0.093	0.067	0.697***	0.086	0.249	0.220	-0.141*	0.071
Baltic	0.070	0.222	-0.440***	0.085	-1.416***	0.214	-0.192	0.229	0.091	0.110
Conservative	Ref.	Ref.	Ref.	Ref.	Ref.	Ref.	Ref.	Ref.	Ref.	Ref.
**Intercept**	-4.582***	0.175	-1.816***	0.087	-1.989***	0.093	-4.275***	0.240	-3.519***	0.099

**p* < 0.05, ***p* < 0.01, ****p* < 0.001 (two-tailed).

Table S3

Multinomial logistic regression analysis of work-family trajectories among women (n = 43,380), multinomial logit coefficients (B) with robust standard errors (RSE) clustered at the country level

	non-worker, partnered parent *vs.* full-time worker, partnered parent		full-time worker, childless single/couple *vs.* full-time worker, partnered parent		self-employed, partnered parent *vs.* full-time worker, partnered parent		part-time worker, partnered parent *vs.* full-time worker, partnered parent		full-time worker, unpartnered parent *vs.* full-time worker, partnered parent	
	B	*RSE*	*B*	*RSE*	*B*	*RSE*	*B*	*RSE*	*B*	*RSE*
**Educational level**										
Low educated	1.779***	0.121	-0.154*	0.063	0.780***	0.151	0.648***	0.097	0.147*	0.072
Moderate educated	0.918***	0.099	-0.091	0.068	0.143	0.078	0.498***	0.108	0.103*	0.049
High educated	Ref.	Ref.	Ref.	Ref.	Ref.	Ref.	Ref.	Ref.	Ref.	Ref.
**Birth cohort**										
Younger cohort	-0.583***	0.116	-0.323***	0.046	-0.238*	0.114	0.380***	0.098	0.163**	0.059
Older cohort	Ref.	Ref.	Ref.	Ref.	Ref.	Ref.	Ref.	Ref.	Ref.	Ref.
**Welfare regime**										
Southern European	0.167	0.267	-0.158	0.177	0.106	0.271	-1.690***	0.311	-1.078***	0.183
Social-democratic	-1.585***	0.252	-0.818***	0.137	-0.799**	0.234	-0.482	0.357	-0.280*	0.116
Eastern European	-1.804***	0.377	-1.147***	0.178	-1.368*	0.623	-3.355***	0.335	-0.701***	0.176
Liberal	0.879***	0.169	-0.105	0.137	-0.043	0.180	-0.241	0.252	-0.505***	0.121
Baltic	-3.276***	0.375	-0.871***	0.148	-2.503***	0.224	-2.798***	0.295	-0.153	0.136
Conservative	Ref.	Ref.	Ref.	Ref.	Ref.	Ref.	Ref.	Ref.	Ref.	Ref.
**Intercept**	-0.592**	0.188	-0.788***	0.146	-1.570***	0.200	-1.139***	0.273	-1.814***	0.157

*$p < 0.05$, **$p < 0.01$, ***$p < 0.001$ (two-tailed).

Table S4

Multilevel multinomial logistic regression analysis of work-family trajectories (*N* = 77,512), multinomial logit coefficients (*B*) and standard errors (*SE*) with shared random effects at the country level

	non-worker, partnered parent *vs.* full-time worker, partnered parent		full-time worker, childless single/couple *vs.* full-time worker, partnered parent		self-employed, partnered parent *vs.* full-time worker, partnered parent		part-time worker, partnered parent *vs.* full-time worker, partnered parent		full-time worker, unpartnered parent *vs.* full-time worker, partnered parent	
	B	*SE*	*B*	*SE*	*B*	*SE*	*B*	*SE*	*B*	*SE*
**Gender**										
Female	3.370***	0.043	0.174***	0.025	-0.001	0.028	2.573***	0.053	1.113***	0.039
Male	Ref.	Ref.	Ref.	Ref.	Ref.	Ref.	Ref.	Ref.	Ref.	Ref.
**Educational level**										
Low educated	1.733***	0.042	0.086*	0.035	0.577***	0.039	0.592***	0.049	0.194***	0.051
Moderate educated	0.826***	0.043	-0.004	0.032	0.046	0.039	0.393***	0.045	0.106*	0.044
High educated	Ref.	Ref.	Ref.	Ref.	Ref.	Ref.	Ref.	Ref.	Ref.	Ref.
**Birth cohort**										
Younger cohort	-0.387***	0.026	0.021	0.026	-0.008	0.029	0.564***	0.039	0.395***	0.040
Older cohort	Ref.	Ref.	Ref.	Ref.	Ref.	Ref.	Ref.	Ref.	Ref.	Ref.
**Welfare regime**										
Southern European	0.086	0.194	-0.275	0.195	0.262	0.195	-1.672***	0.201	-1.248***	0.206
Social-democratic	-1.481***	0.243	-0.525*	0.241	-0.448	0.242	-0.419	0.242	-0.200	0.244
Eastern European	-1.610***	0.181	-0.913***	0.181	-1.100***	0.183	-3.039***	0.191	-0.690***	0.184
Liberal	0.888*	0.385	-0.143	0.396	0.354	0.392	-0.200	0.408	-0.407	0.447
Baltic	-2.833***	0.250	-0.637**	0.242	-1.941***	0.252	-2.382***	0.251	-0.040	0.243
Conservative	Ref.	Ref.	Ref.	Ref.	Ref.	Ref.	Ref.	Ref.	Ref.	Ref.
**Intercept**	-4.079***	0.142	-1.400***	0.135	-1.779***	0.136	-3.842***	0.147	-3.121***	0.142
**Random effects**										
	B	*SE*	95% Confidence Interval							
Country-level variance	0.118	0.032	[0.069, 0.202]							

*$p < 0.05$, **$p < 0.01$, ***$p < 0.001$ (two-tailed).

Table S5

Multilevel multinomial logistic regression analysis of work-family trajectories among men (n = 34,132), multinomial logit coefficients (B) and standard errors (SE) with shared random effects at the country level

	non-worker, partnered parent *vs.* full-time worker, partnered parent		full-time worker, childless single/couple *vs.* full-time worker, partnered parent		self-employed, partnered parent *vs.* full-time worker, partnered parent		part-time worker, partnered parent *vs.* full-time worker, partnered parent		full-time worker, unpartnered parent *vs.* full-time worker, partnered parent	
	B	*SE*	*B*	*SE*	*B*	*SE*	*B*	*SE*	*B*	*SE*
**Educational level**										
Low educated	1.212***	0.128	0.250***	0.049	0.391***	0.050	0.078	0.133	0.197*	0.093
Moderate educated	0.201	0.134	0.097*	0.045	0.011	0.050	-0.197	0.120	0.151	0.081
High educated	Ref.	Ref.	Ref.	Ref.	Ref.	Ref.	Ref.	Ref.	Ref.	Ref.
**Birth cohort**										
Younger cohort	0.440***	0.086	0.278***	0.036	0.145***	0.037	0.973***	0.117	0.803***	0.075
Older cohort	Ref.	Ref.	Ref.	Ref.	Ref.	Ref.	Ref.	Ref.	Ref.	Ref.
**Welfare regime**										
Southern European	-0.016	0.187	-0.230	0.151	0.496**	0.150	-0.789***	0.212	-1.324***	0.192
Social-democratic	-0.412	0.250	-0.136	0.184	-0.029	0.185	0.114	0.223	0.089	0.199
Eastern European	0.301	0.171	-0.576***	0.140	-0.784***	0.143	-1.078***	0.199	-0.614***	0.157
Liberal	1.187**	0.414	-0.098	0.338	0.692*	0.315	0.244	0.536	-0.147	0.476
Baltic	0.068	0.241	-0.442*	0.189	-1.419***	0.210	-0.196	0.242	0.089	0.203
Conservative	Ref.	Ref.	Ref.	Ref.	Ref.	Ref.	Ref.	Ref.	Ref.	Ref.
**Intercept**	-4.600***	0.170	-1.826***	0.107	-2.011***	0.109	-4.284***	0.169	-3.522***	0.134
**Random effects**										
	B	*SE*	95% Confidence Interval							
Country-level variance	0.064	0.019	[0.036, 0.114]							

*p < 0.05, **p < 0.01, ***p < 0.001 (two-tailed).

Table S6

Multilevel multinomial logistic regression analysis of work-family trajectories among women (n = 43,380), multinomial logit coefficients (B) and standard errors (SE) with shared random effects at the country level

	non-worker, partnered parent *vs.* full-time worker, partnered parent		full-time worker, childless single/couple *vs.* full-time worker, partnered parent		self-employed, partnered parent *vs.* full-time worker, partnered parent		part-time worker, partnered parent *vs.* full-time worker, partnered parent		full-time worker, unpartnered parent *vs.* full-time worker, partnered parent	
	B	*SE*	*B*	*SE*	*B*	*SE*	*B*	*SE*	*B*	*SE*
**Educational level**										
Low educated	1.787***	0.046	-0.147**	0.052	0.789***	0.063	0.656***	0.054	0.151*	0.061
Moderate educated	0.865***	0.046	-0.130**	0.047	0.096	0.065	0.455***	0.050	0.074	0.054
High educated	Ref.	Ref.	Ref.	Ref.	Ref.	Ref.	Ref.	Ref.	Ref.	Ref.
**Birth cohort**										
Younger cohort	-0.602***	0.029	-0.352***	0.039	-0.260***	0.046	0.373***	0.043	0.133**	0.047
Older cohort	Ref.	Ref.	Ref.	Ref.	Ref.	Ref.	Ref.	Ref.	Ref.	Ref.
**Welfare regime**										
Southern European	-0.040	0.300	-0.377	0.303	-0.105	0.303	-1.909***	0.305	-1.296***	0.311
Social-democratic	-1.919***	0.372	-1.154**	0.374	-1.133**	0.376	-0.811*	0.371	-0.612	0.375
Eastern European	-2.066***	0.279	-1.407***	0.281	-1.633***	0.283	-3.625***	0.287	-0.969**	0.282
Liberal	0.631	0.586	-0.357	0.610	-0.294	0.625	-0.491	0.604	-0.755	0.662
Baltic	-3.466***	0.379	-1.065**	0.373	-2.696***	0.390	-2.987***	0.380	-0.348	0.373
Conservative	Ref.	Ref.	Ref.	Ref.	Ref.	Ref.	Ref.	Ref.	Ref.	Ref.
**Intercept**	-0.318	0.208	-0.510*	0.208	-1.294***	0.213	-0.874***	0.210	-1.538***	0.212
**Random effects**										
	B	*SE*	95% Confidence Interval							
Country-level variance	0.284	0.078	[0.165, 0.487]							

*p < 0.05, **p < 0.01, ***p < 0.001 (two-tailed).

Table S7

Multinomial logistic regression analysis of work-family trajectories with four birth cohorts (*N* = 77,512), average marginal effects

	full-time worker, partnered parent	non-worker, partnered parent	full-time worker, childless single/couple	self-employed, partnered parent	part-time worker, partnered parent	full-time worker, unpartnered parent
**Gender**						
Female	-0.249***	0.239***	-0.037***	-0.055***	0.073***	0.028***
Male	Ref.	Ref.	Ref.	Ref.	Ref.	Ref.
**Educational level**						
Low educated	-0.137***	0.139***	-0.024***	0.020	0.007	-0.004
Moderate educated	-0.058***	0.059***	-0.010	-0.003	0.011*	0.000
High educated	Ref.	Ref.	Ref.	Ref.	Ref.	Ref.
**Birth cohort**						
Before 1940	Ref.	Ref.	Ref.	Ref.	Ref.	Ref.
1940-1945	0.038***	-0.039***	-0.007	-0.019**	0.014***	0.012**
1946-1950	0.043***	-0.058***	-0.006	-0.018*	0.022***	0.017***
After 1950	0.003	-0.066***	0.001	-0.003	0.039***	0.025***
**Welfare regime**						
Southern European	0.017	0.059**	-0.010	0.047*	-0.082***	-0.031***
Social-democratic	0.118***	-0.114***	-0.017**	-0.007	0.008	0.012*
Eastern European	0.264***	-0.099***	-0.033**	-0.036	-0.095***	-0.001
Liberal	-0.074**	0.133***	-0.026***	0.025***	-0.037*	-0.021***
Baltic	0.286***	-0.165***	-0.010	-0.067***	-0.085***	0.041***
Conservative	Ref.	Ref.	Ref.	Ref.	Ref.	Ref.

*$p < 0.05$, **$p < 0.01$, ***$p < 0.001$ (two-tailed).
