# Supplementary material for: Work-family trajectories across Europe: differences between social groups and welfare regimes
Source: Front Sociol. 2023 Nov 30;8:1100700. doi: 10.3389/fsoc.2023.1100700 (PMC10720591; doi:10.3389/fsoc.2023.1100700)
Supplement: Supplementary file 2 [file Data_Sheet_2.pdf]

Firat M, Visser M and Kraaykamp G (2023) Work-family trajectories across Europe: Differences between social groups and welfare regimes. *Front. Sociol.* 8:1100700. doi: 10.3389/fsoc.2023.1100700

Appendix

Figure A1. Cut-off criteria for the Ward hierarchical clustering of multichannel sequence distances

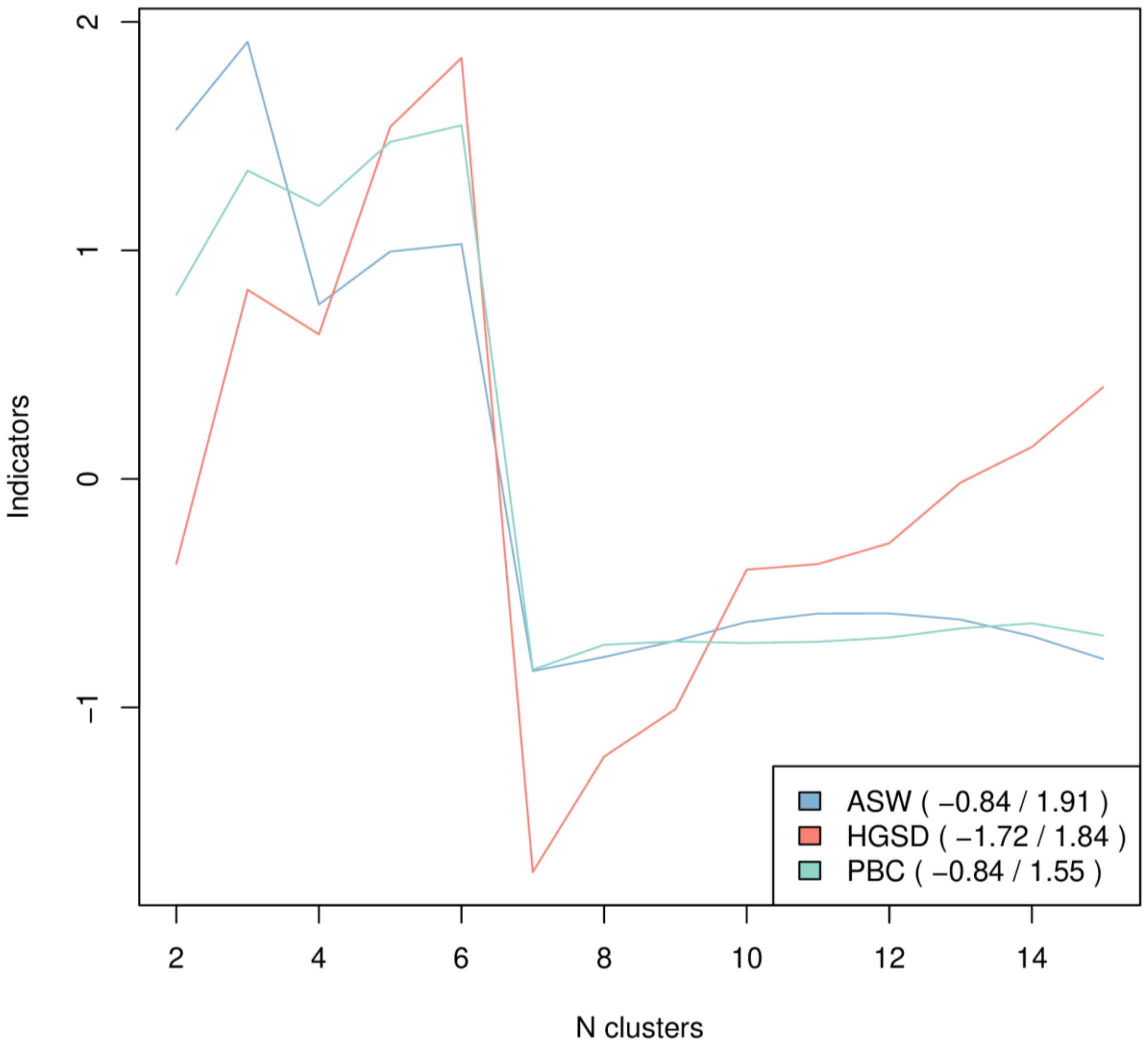

Note: Because the scales for the ASW (Average Silhouette Width), HGSD (Hubert's Gamma Somers' D) and the PBC (Point Biserial Correlation) are different from one another, the values displayed in the figure are standardized (*z*-scores). Higher values indicate better cluster solutions.

Table A1.1

User-defined substitution cost matrix for transitions between work states (indel costs = 5)

	disability	full-time employment	missing	non-employment	part-time employment	self-employment	unemployment
disability	0	10	5	6	9	10	7
full-time employment	10	0	5	10	8	9	10
missing	5	5	0	5	5	5	5
non-employment	6	10	5	0	9	9	6
part-time employment	9	8	5	9	0	7	10
self-employment	10	9	5	9	7	0	10
unemployment	7	10	5	6	10	10	0

Table A1.2

User-defined substitution cost matrix for transitions between family states (indel costs = 5)

	partnered, children	partnered, no children	single, children	single, no children	unpartnered, children	unpartnered, no children
partnered, children	0	7	6	6	10	10
partnered, no children	7	0	6	6	10	10
single, children	6	6	0	6	5	5
single, no children	6	6	6	0	5	5
unpartnered, children	10	10	5	5	0	7
unpartnered, no children	10	10	5	5	7	0

Table A2

Frequencies (row percentages) of work-family trajectories by gender, educational level, birth cohort and welfare regime

	full-time worker, partnered parent (*n* = 43,615)	non-worker, partnered parent (*n* = 12,031)	full-time worker, childless single/couple (*n* = 7,793)	self-employed, partnered parent (*n* = 6,391)	part-time worker, partnered parent (*n* = 4,071)	full-time worker, unpartnered parent (*n* = 3,611)
**Gender**						
Female	44.99	94.68	46.41	39.65	89.49	71.20
Male	55.01	5.32	53.59	60.35	10.51	28.80
**Educational level**						
Low educated	30.79	65.49	32.50	48.22	31.88	27.33
Moderate educated	44.98	26.97	42.38	32.56	43.55	47.41
High educated	24.23	7.54	25.11	19.21	24.56	25.26
**Birth cohort**						
Younger cohort	61.71	46.90	60.72	56.77	70.52	70.15
Older cohort	38.29	53.10	39.28	43.23	29.48	29.85
**Welfare regime**						
Southern European	16.06	39.84	20.34	36.99	9.09	6.73
Social-democratic	11.94	4.78	11.48	11.13	22.11	14.23
Eastern European	34.36	16.37	22.43	16.96	6.31	28.88
Conservative	24.09	35.64	34.49	31.34	57.18	29.69
Liberal	0.57	1.70	0.76	1.14	1.01	0.47
Baltic	12.99	1.67	10.50	2.44	4.30	19.99

Table A3

Frequencies (column percentages) of work-family trajectories across the countries within welfare regimes

	full-time worker, partnered parent	non-worker, partnered parent	full-time worker, childless single/couple	self-employed, partnered parent	part-time worker, partnered parent	full-time worker, unpartnered parent
Southern European (n = 16,358)	***42.81***	***29.30***	***9.69***	***14.45***	***2.26***	***1.49***
Cyprus (*n* = 1,090)	52.84	29.63	3.49	10.18	2.29	1.56
Greece (*n* = 3,470)	32.42	32.65	8.79	23.43	0.92	1.79
Italy (*n* = 4,939)	44.50	27.70	9.41	13.61	3.16	1.62
Malta (*n* = 1,137)	29.46	43.89	17.85	6.07	2.29	0.44
Portugal (*n* = 1,004)	61.16	17.13	9.66	8.76	1.49	1.79
Spain (*n* = 4,718)	45.68	27.51	10.11	12.95	2.46	1.29
Social-democratic (n = 8,801)	***59.15***	***6.53***	***10.17***	***8.08***	***10.23***	***5.84***
Denmark (*n* = 3,601)	56.48	7.44	9.91	9.03	11.91	5.22
Finland (*n* = 1,706)	65.12	5.04	10.84	8.73	3.28	6.98
Sweden (*n* = 3,494)	58.99	6.33	10.10	6.78	11.88	5.92
Eastern European (n = 21,086)	***71.07***	***9.34***	***8.29***	***5.14***	***1.22***	***4.95***
Bulgaria (*n* = 1,729)	75.01	4.22	11.16	2.78	1.74	5.09
Croatia (*n* = 1,992)	65.86	19.78	7.28	2.86	0.60	3.61
Czechia (*n* = 4,453)	78.71	1.59	7.07	2.25	2.11	8.26
Hungary (*n* = 1,351)	77.13	5.40	8.07	3.03	1.04	5.33
Poland (*n* = 4,728)	62.86	11.19	6.09	14.42	1.54	3.89
Romania (*n* = 1,746)	65.69	21.13	6.87	1.60	0.92	3.78
Slovakia (*n* = 1,901)	74.54	3.89	14.94	2.63	0.47	3.52
Slovenia (*n* = 3,186)	71.97	12.12	9.23	2.45	0.28	3.95
Conservative (n = 22,884)	***45.91***	***18.74***	***11.75***	***8.75***	***10.17***	***4.68***
Austria (*n* = 3,244)	45.68	18.87	11.56	10.30	7.31	6.29
Belgium (*n* = 5,494)	47.34	18.67	12.65	8.57	7.72	5.04
France (*n* = 4,007)	49.84	17.59	10.56	9.68	6.41	5.91
Germany (*n* = 4,392)	52.98	13.98	10.52	6.53	12.07	3.92
Luxembourg (*n* = 1,122)	46.17	23.80	12.66	6.60	6.68	4.10
Switzerland (*n* = 2,653)	33.55	20.13	13.87	11.38	17.94	3.13
The Netherlands (n = 1,972)	34.99	26.88	11.31	7.45	16.68	2.69
Liberal (Ireland; n = 645)	***38.76***	***31.78***	***9.15***	***11.32***	***6.36***	***2.64***
Baltic (n = 7,738)	***73.22***	***2.60***	***10.57***	***2.02***	***2.26***	***9.33***
Estonia (*n* = 4,458)	74.34	1.75	10.57	2.53	2.33	8.48
Latvia (*n* = 1,438)	71.49	2.85	11.89	0.97	1.18	11.61
Lithuania (*n* = 1,842)	71.88	4.45	9.55	1.57	2.93	9.61

Table A4

Multinomial logistic regression analysis of work-family trajectories (N = 77,512), average marginal effects

	full-time worker, partnered parent	non-worker, partnered parent	full-time worker, childless single/couple	self-employed, partnered parent	part-time worker, partnered parent	full-time worker, unpartnered parent
**Gender**						
Female	-0.250***	0.239***	-0.037***	-0.054***	0.074***	0.028***
Male	Ref.	Ref.	Ref.	Ref.	Ref.	Ref.
**Educational level**						
Low educated	-0.137***	0.141***	-0.023***	0.020	0.004	-0.006
Moderate educated	-0.058***	0.059***	-0.009	-0.003	0.011*	0.000
High educated	Ref.	Ref.	Ref.	Ref.	Ref.	Ref.
**Birth cohort**						
Younger cohort	-0.001	-0.047***	0.002	0.000	0.028***	0.017***
Older cohort	Ref.	Ref.	Ref.	Ref.	Ref.	Ref.
**Welfare regime**						
Southern European	0.018	0.057*	-0.010	0.047*	-0.081***	-0.031***
Social-democratic	0.119***	-0.115***	-0.017**	-0.007	0.009	0.012*
Eastern European	0.264***	-0.101***	-0.033**	-0.036	-0.094***	0.000
Liberal	-0.072**	0.134***	-0.026***	0.024***	-0.038*	-0.021***
Baltic	0.285***	-0.166***	-0.010	-0.067***	-0.084***	0.041***
Conservative	Ref.	Ref.	Ref.	Ref.	Ref.	Ref.

*$p < 0.05$, **$p < 0.01$, ***$p < 0.001$ (two-tailed).

Table A5

Multinomial logistic regression analysis of work-family trajectories among men (*n* = 34,132) and women (*n* = 43,380), average marginal effects

	full-time worker, partnered parent		non-worker, partnered parent		full-time worker, childless single/couple		self-employed, partnered parent		part-time worker, partnered parent		full-time worker, unpartnered parent	
	men	women	men	women	men	women	men	women	men	women	men	women
**Educational level**												
Low educated	**-0.065*****	**-0.181*****	**0.021*****	**0.236*****	**0.014**	**-0.060*****	0.028*	0.012	-0.001	0.006	**0.002**	**-0.014****
Moderate educated	**-0.016**	**-0.083*****	**0.002**	**0.104*****	**0.011**	**-0.032*****	0.001	-0.006	**-0.003**	**0.021****	0.004	-0.004
High educated	Ref.	Ref.	Ref.	Ref.	Ref.	Ref.	Ref.	Ref.	Ref.	Ref.	Ref.	Ref.
**Birth cohort**												
Younger cohort	**-0.062*****	**0.052****	**0.006*****	**-0.091*****	**0.022*****	**-0.016*****	0.005	-0.002	0.010***	0.042***	**0.019*****	**0.015*****
Older cohort	Ref.	Ref.	Ref.	Ref.	Ref.	Ref.	Ref.	Ref.	Ref.	Ref.	Ref.	Ref.
**Welfare regime**												
Southern European	-0.029	0.047	**0.001**	**0.105****	-0.021	-0.001	**0.086***	**0.018**	**-0.009***	**-0.137*****	-0.028***	-0.033***
Social-democratic	**0.014**	**0.202*****	**-0.005***	**-0.201*****	-0.015*	-0.021**	**-0.001**	**-0.012**	0.003	0.015	0.005	0.017
Eastern European	**0.110*****	**0.381*****	**0.010**	**-0.186*****	-0.047***	-0.022*	-0.051*	-0.024	**-0.010***	**-0.160*****	**-0.013****	**0.011**
Liberal	-0.080***	-0.064*	0.030***	0.216***	-0.029***	-0.029***	**0.086*****	**-0.021****	**0.002**	**-0.071****	**-0.009*****	**-0.029*****
Baltic	**0.105*****	**0.425*****	**0.004**	**-0.297*****	**-0.037*****	**0.007**	**-0.081*****	**-0.053*****	**-0.001**	**-0.147*****	**0.010***	**0.065*****
Conservative	Ref.	Ref.	Ref.	Ref.	Ref.	Ref.	Ref.	Ref.	Ref.	Ref.	Ref.	Ref.

p* < 0.05, ***p* < 0.01, ****p* < 0.001 (two-tailed). **Bold coefficients indicate a statistically significant difference in effects for men and women based on logistic regressions.
